# Supplementary figures and images for: Validation and tuning of in situ transcriptomics image processing workflows with crowdsourced annotations
Source: PLoS Comput Biol. 2021 Aug 9;17(8):e1009274. doi: 10.1371/journal.pcbi.1009274 (PMC8376178; doi:10.1371/journal.pcbi.1009274)

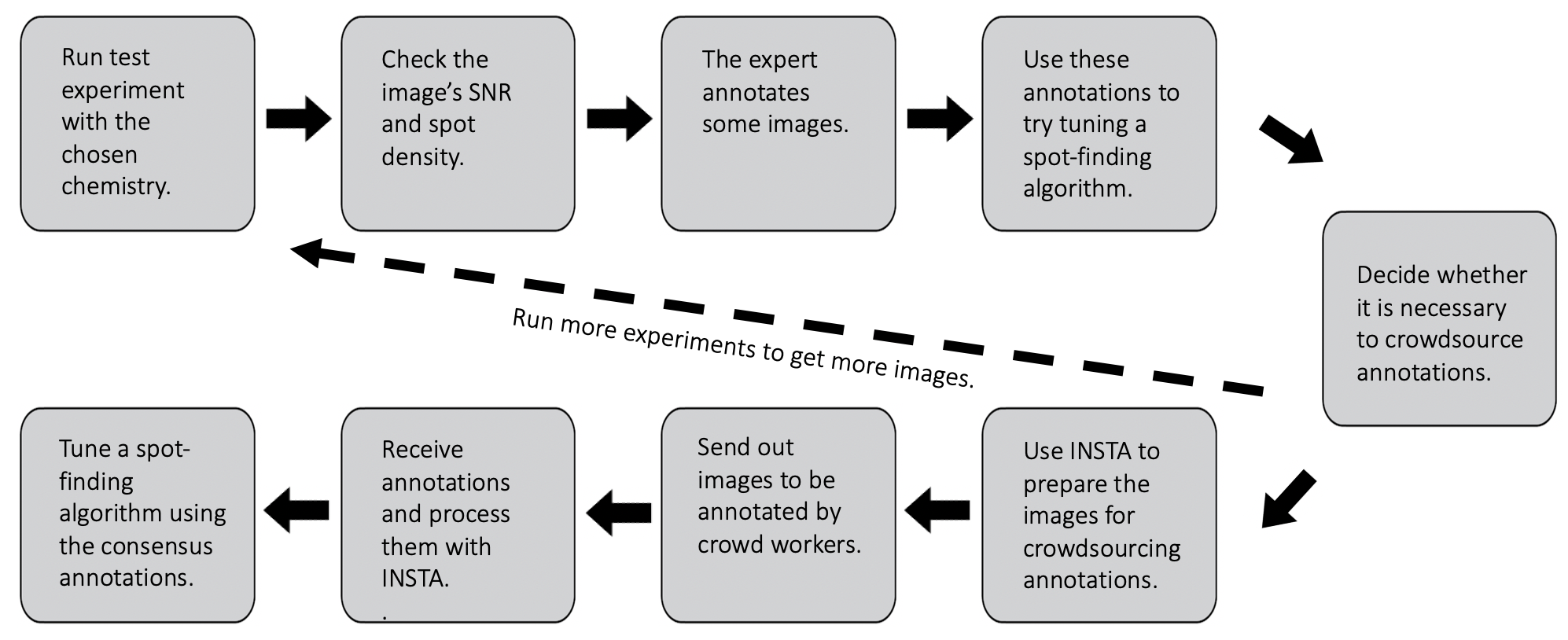

Supplement: S1 Fig — (TIF) [file pcbi.1009274.s001.tif]

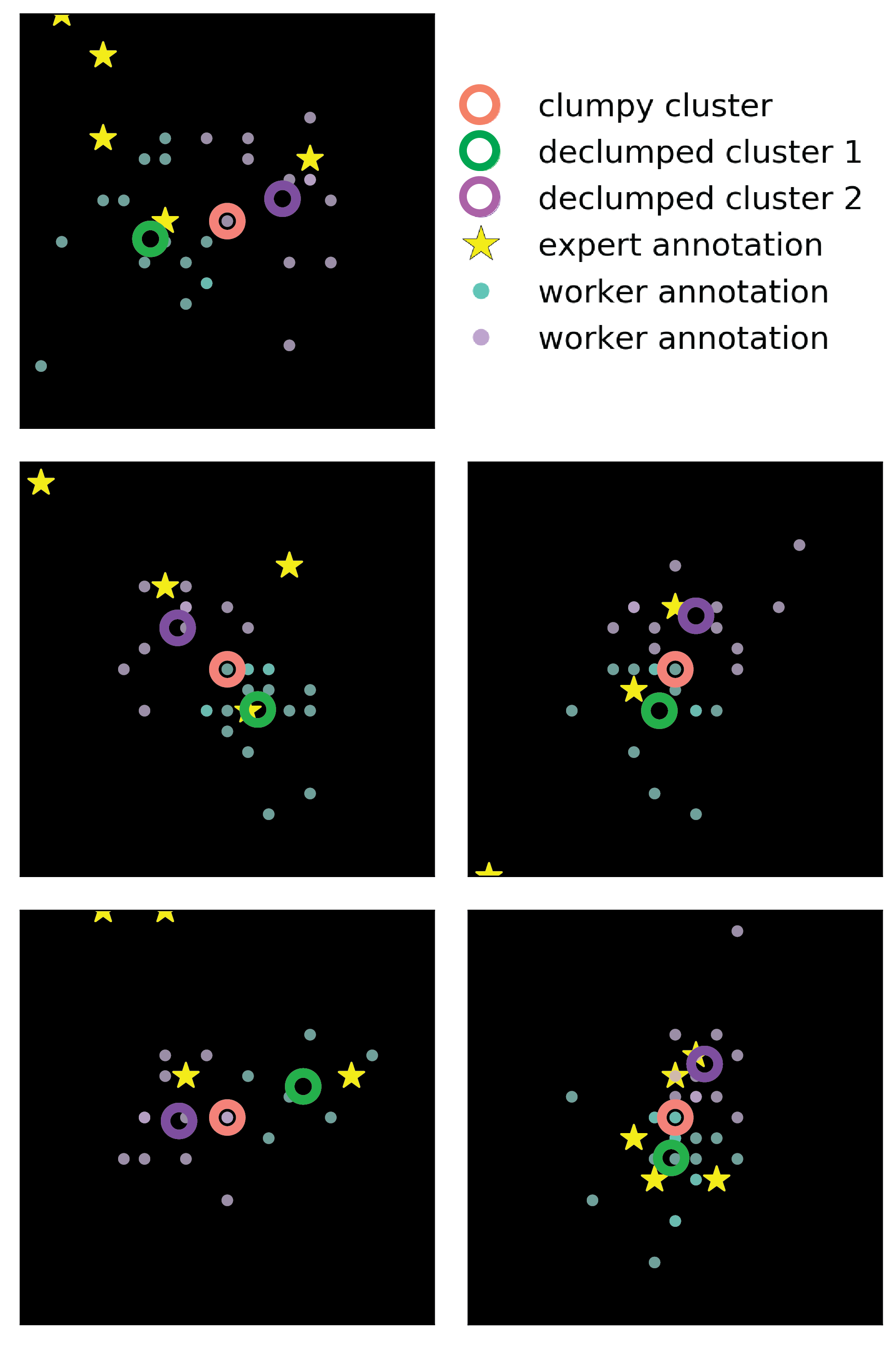

Supplement: S2 Fig — Shown here with synthetic spot images generated by the SpotImage tool on mouse lung image background. (Image background omitted here for clarity of figure). (TIF) [file pcbi.1009274.s002.tif]

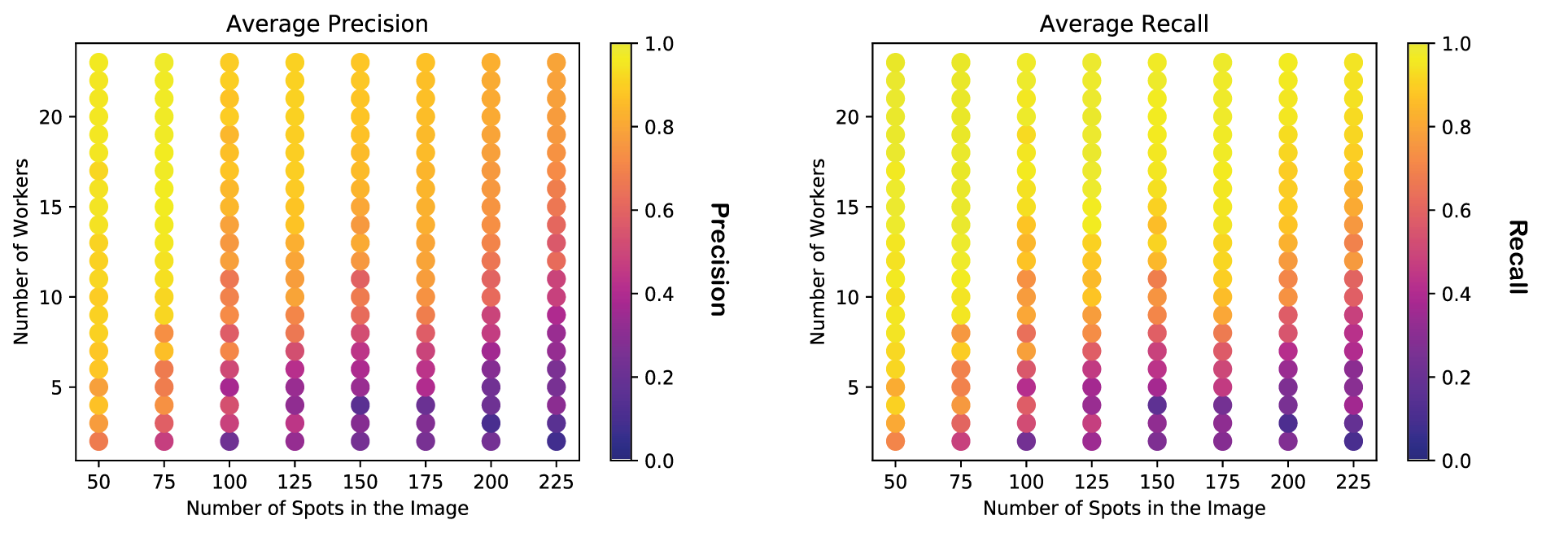

Supplement: S3 Fig — Using simulated spot images, we found that at least 20 workers are necessary to consistently yield precision and recall greater than 95% for images which contain 75 spots, and that the number of workers required for reliable annotation of an image does not increase dramatically as the number of spots in the image increases. All spot images used for this analysis were simulated with spots of SNR = 10 over mouse lung tissue background images. Each marker value represents the average across 10 groups of workers. (TIF) [file pcbi.1009274.s003.tif]

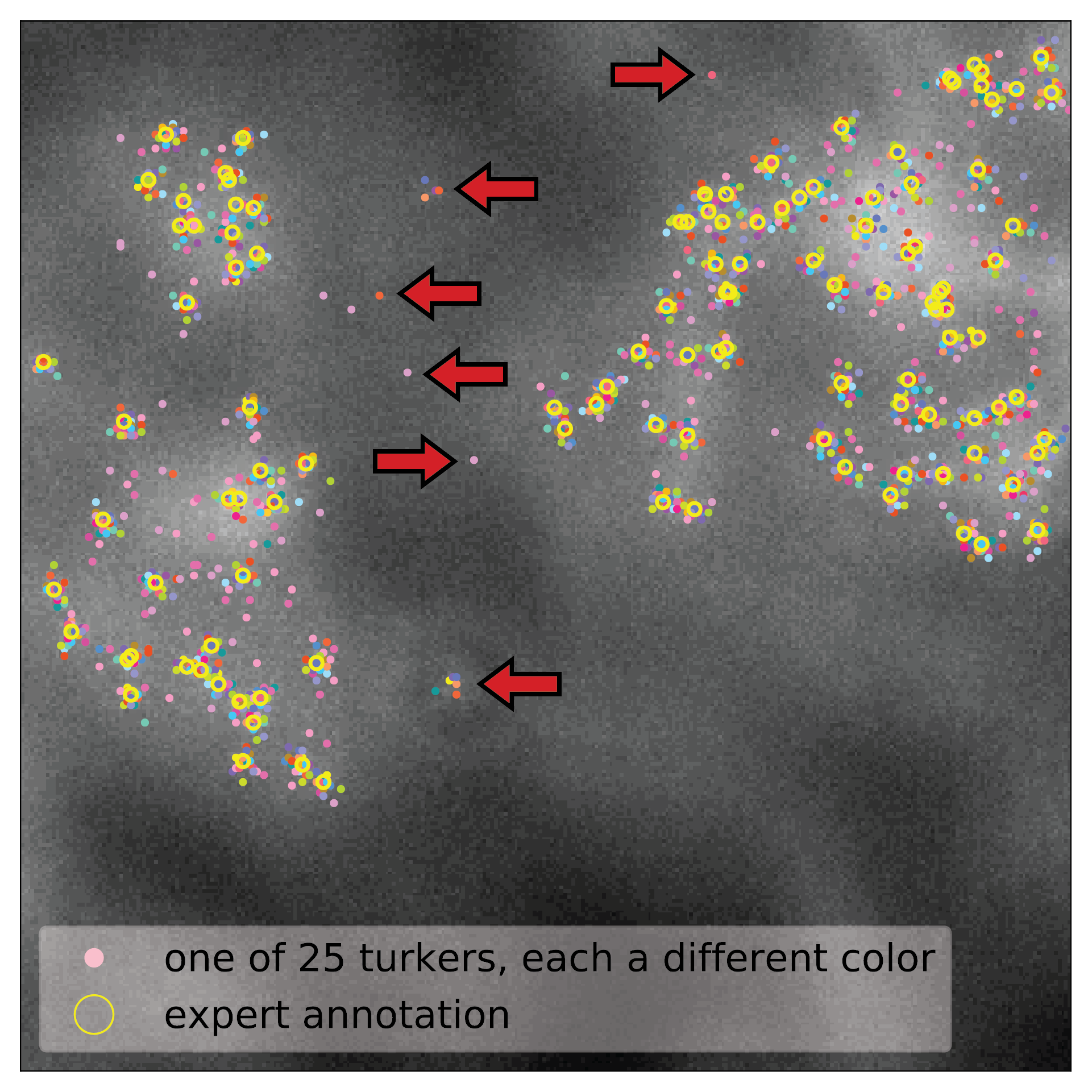

Supplement: S4 Fig — (TIF) [file pcbi.1009274.s004.tif]

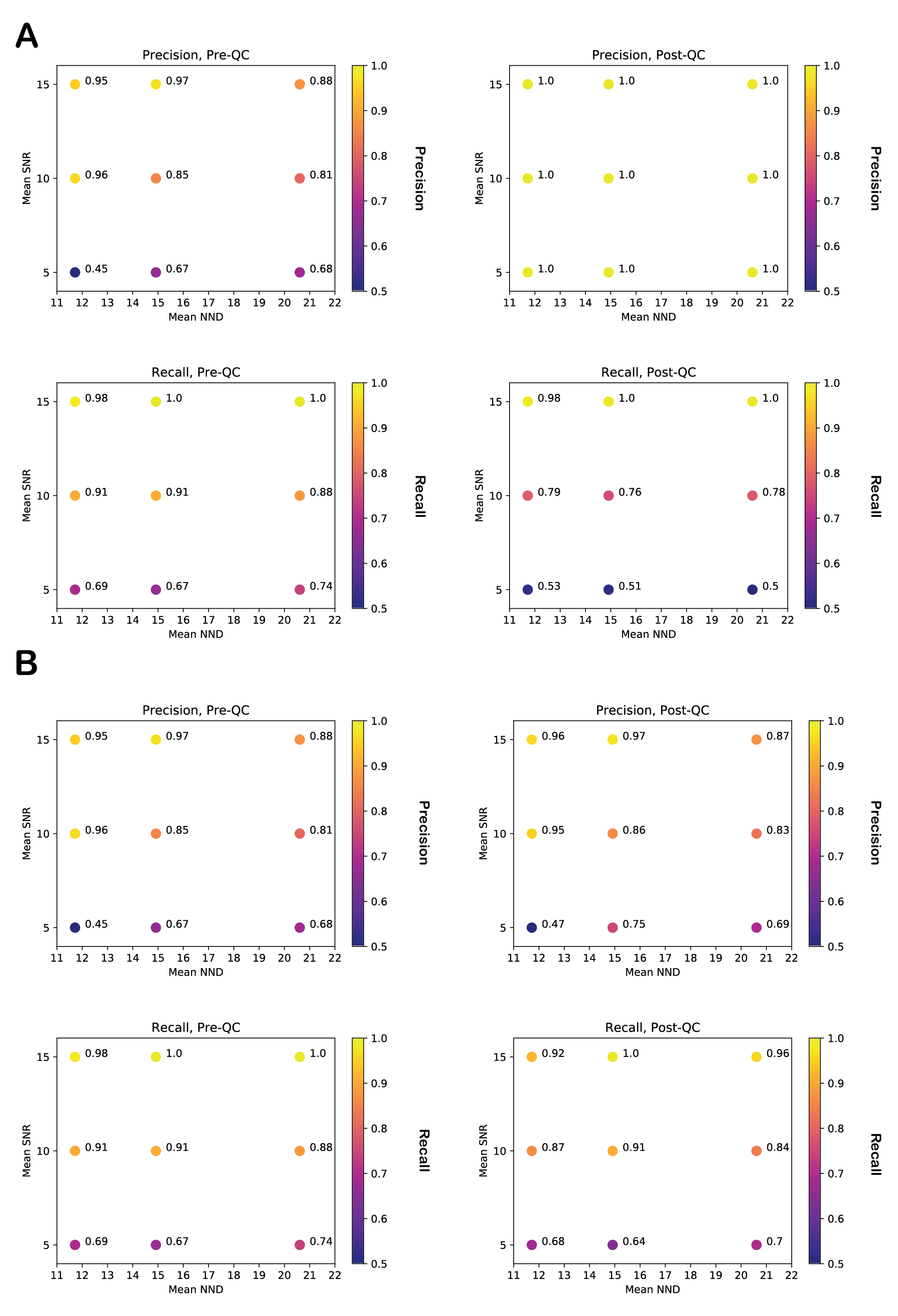

Supplement: S5 Fig — (A) Thresholding clusters by the number of annotations in the cluster improved precision by 17% while decreasing recall by 10% on average in an experiment with images of mean SNR = 5, 10, and 15 and average NND = ~ 11, 15, and 19. (B) Thresholding clusters by the fraction of unique workers who contribute multiple times to the cluster improved recall by 2% while decreasing precision by 1% on average in an experiment with images of mean SNR = 5, 10, and 15 and average NND = ~ 11.5, 15, and 20.5. (TIF) [file pcbi.1009274.s005.tif]

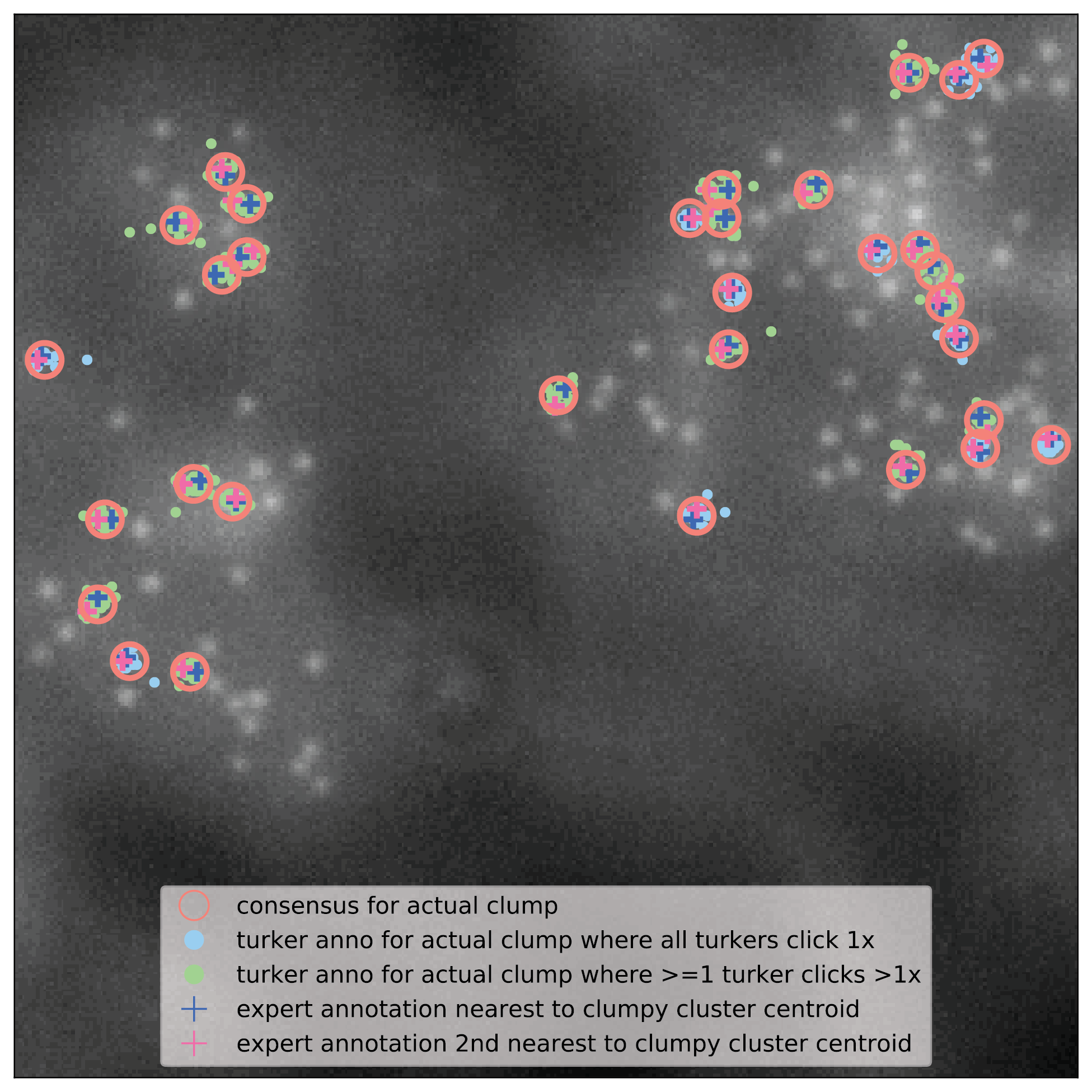

Supplement: S6 Fig — Markers are only shown for actual clumps. (TIF) [file pcbi.1009274.s006.tif]

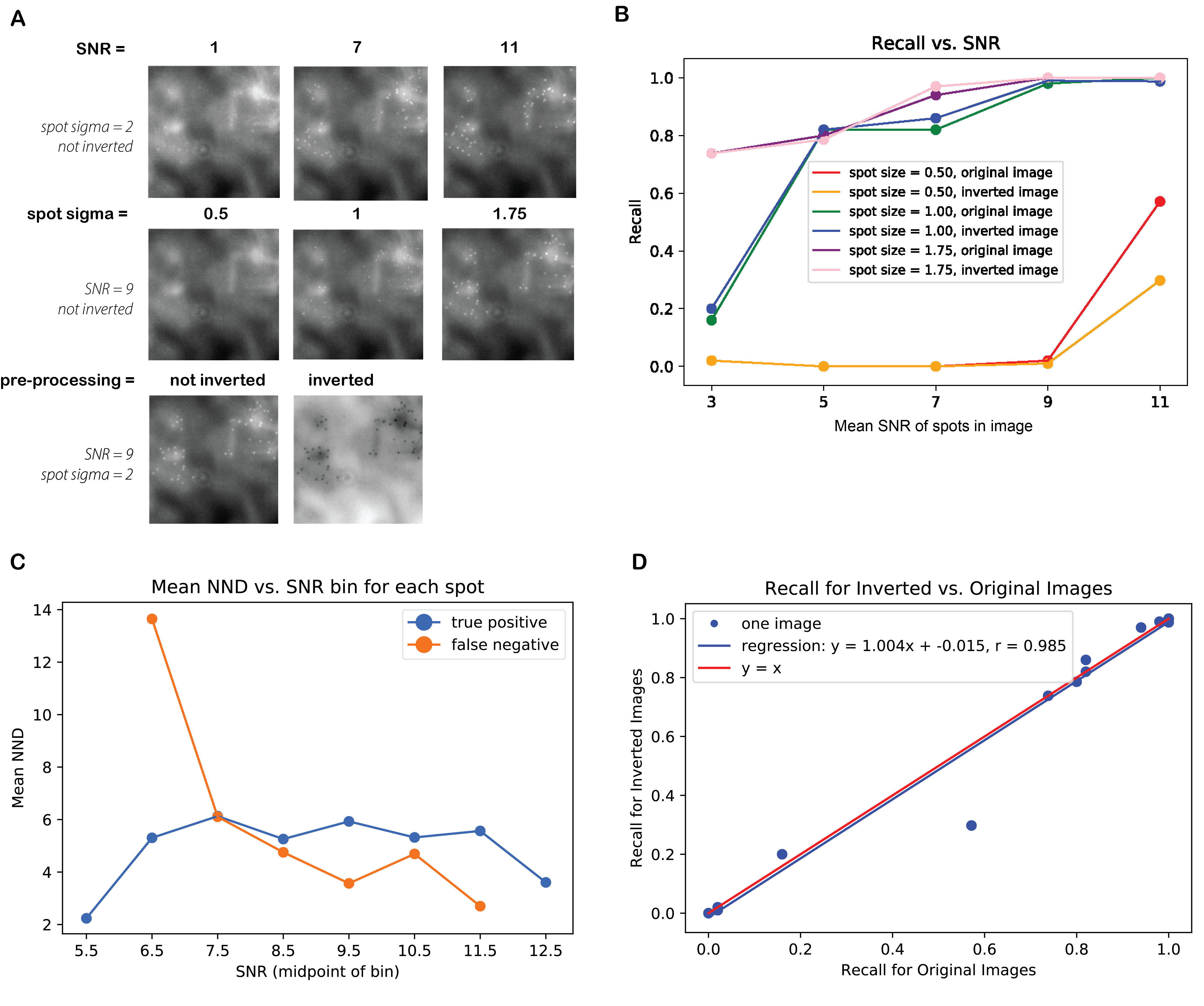

Supplement: S7 Fig — (A) This experiment used synthetic images with spot SNR ranging from 1 to 11; spot size = 0.5, 1.0, and 1.75; plus inverted and not inverted. A subset of these images are shown here. (B) In an experiment using these images, small spot sizes required larger mean SNR to achieve good recall. (C) At lower SNR values, even spots with large nearest neighbor distances tended to be missed, and as spot SNR increased, the median NND of undetected spots decreased. (D) A linear regression between recall with inversion and recall without inversion resulted in a slope of 1.004 with Pearson’s correlation coefficient of r = 0.985. In other words, there was insufficient evidence that inverting the images improved worker performance. (TIF) [file pcbi.1009274.s007.tif]

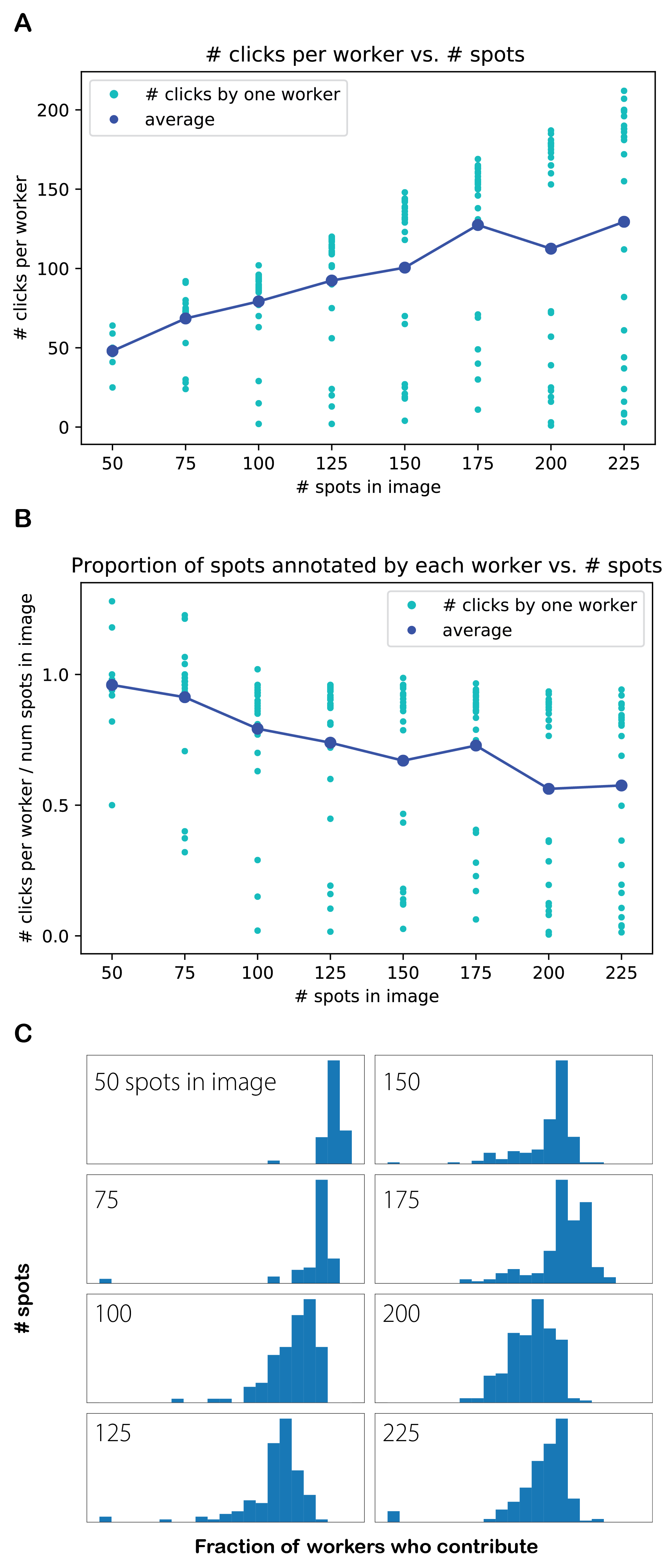

Supplement: S8 Fig — (A) The number of clicks per worker per image increased as the number of spots in the image increased until it leveled off around 120 on average, suggesting that 120 was the upper bound on the number of spots workers were willing to click. (B) As the number of spots increased, the fraction of spots that workers were willing to click decreased. On average, workers annotated almost all spots for images with 50 spots but only about 60% of all spots for images with 200 spots. (C) Even though the workers annotated a smaller fraction of the spots as the number of spots in the image increased, most spots were still getting annotations from at least half the workers. (TIF) [file pcbi.1009274.s008.tif]

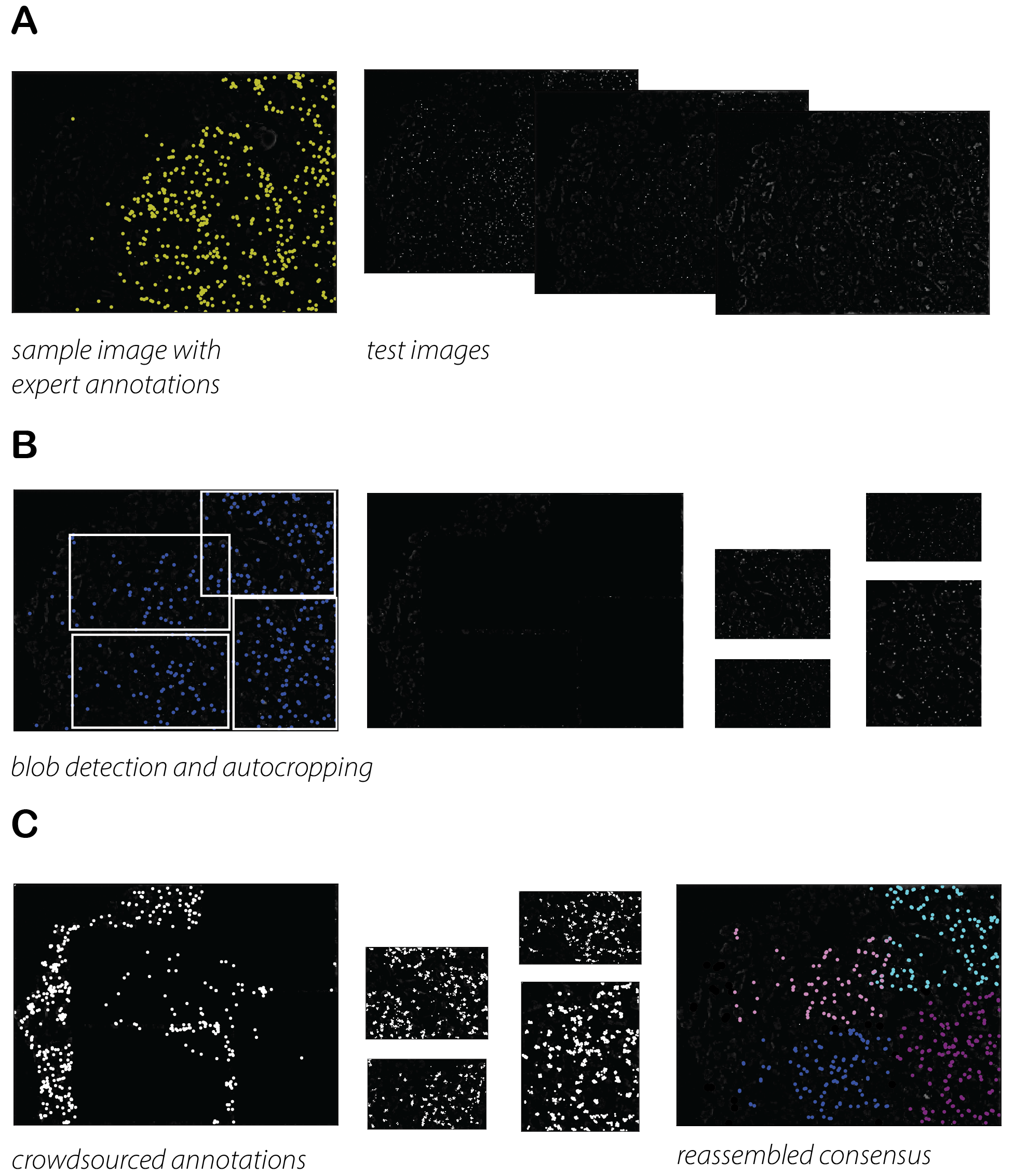

Supplement: S9 Fig — (A) The inputs to the pipeline were one sample image with the RCA chemistry, expert spot location annotations for that image, and three test images without annotations. (B) Blob detection provided a general idea of the regions where spots were located and images were automatically subdivided. (C) Annotations were crowdsourced, QC’d, and reassembled as consensus. (TIF) [file pcbi.1009274.s009.tif]

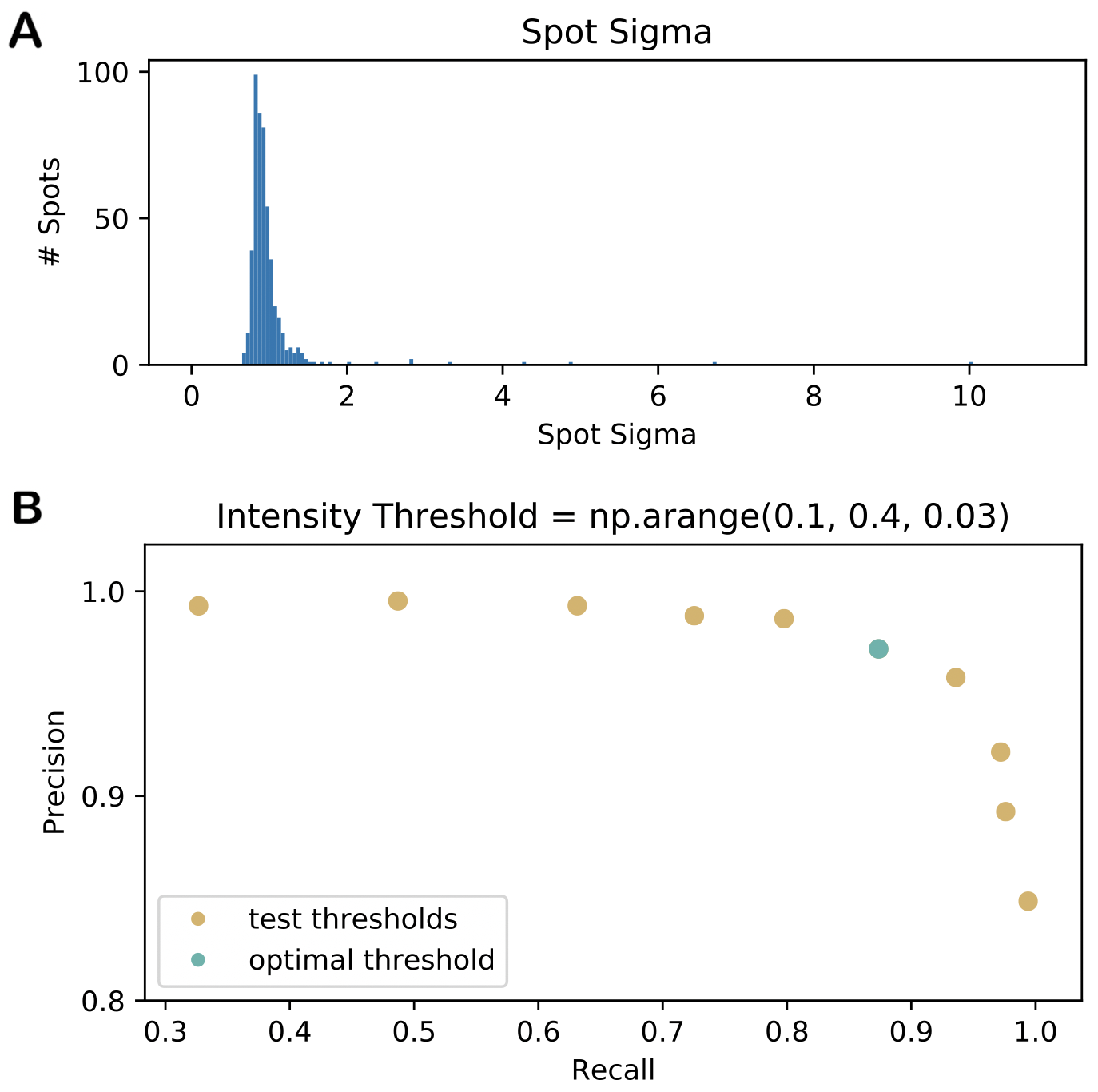

Supplement: S10 Fig — (A) The largest sigma associated with a spot annotated by the “expert” is designated sigma_max, which indicates the maximum spot size of a spot that can be detected. (B) The threshold parameter, which indicates the lower bound on the brightness of a detected spot, is chosen which optimizes precision times recall when blob_log() is executed on the sample image. (TIF) [file pcbi.1009274.s010.tif]

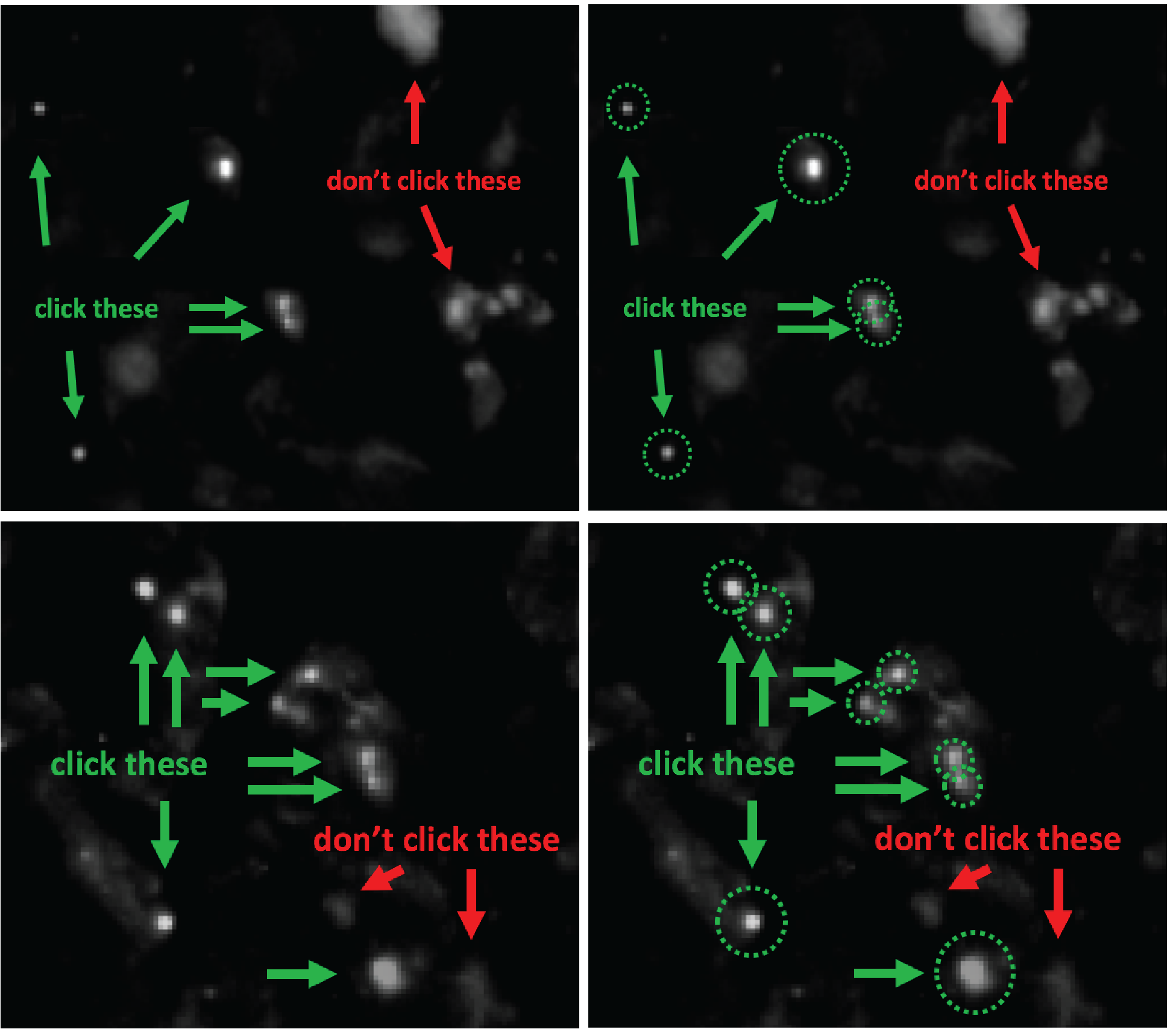

Supplement: S11 Fig — Top and bottom rows: design variants 1 and 2, respectively. Left and right columns: with and without circles drawn around correct spots. (TIF) [file pcbi.1009274.s011.tif]

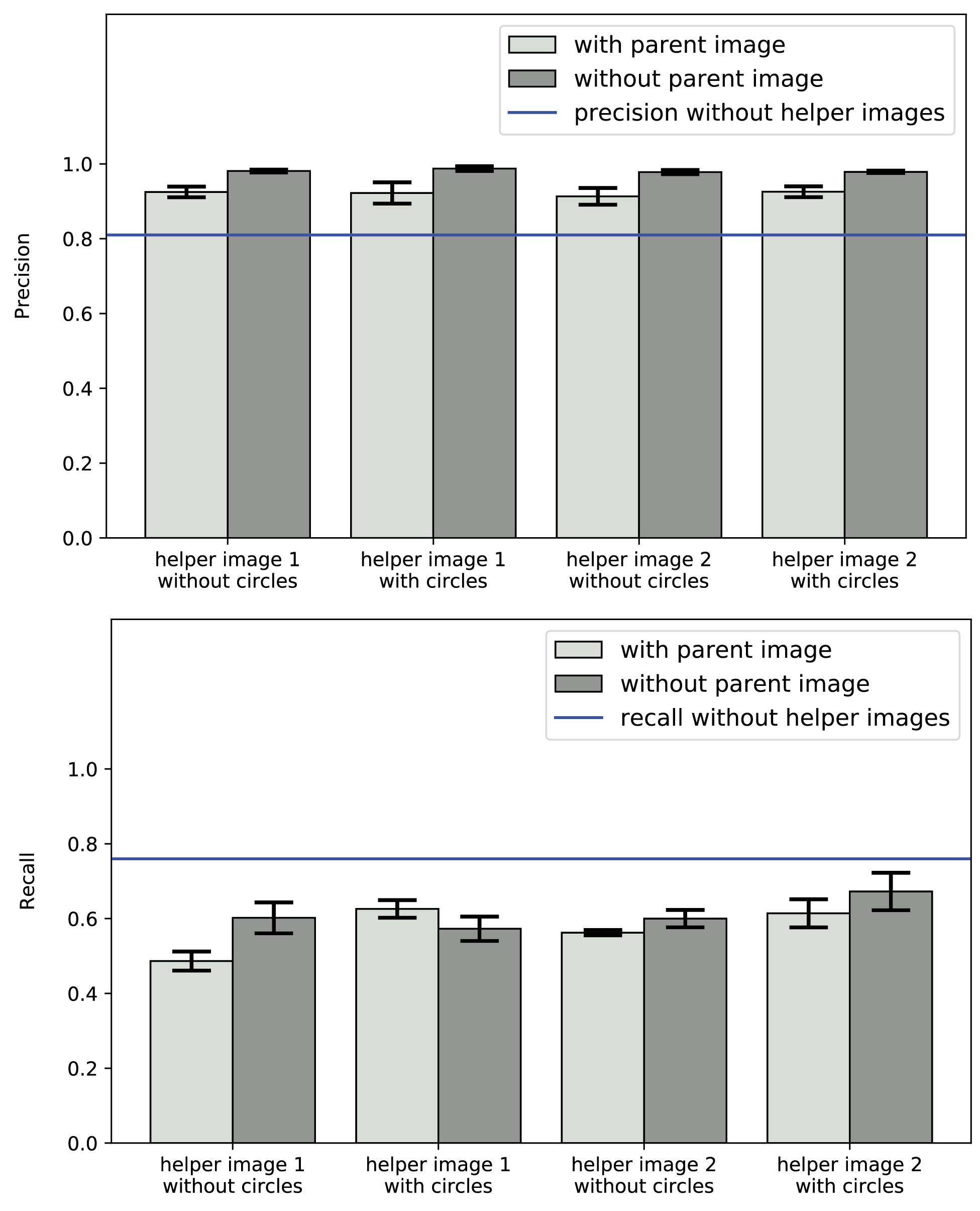

Supplement: S12 Fig — On average, the inclusion of helper images For RCA image ISS_rnd1_ch1_z0, which contains 287 spots, the inclusion of helper images on average increased precision by 14% (95% with helper images and 81% without) and decreased recall by 16% (59% with helper images and 76% without). When the spots in the helper image were circled, precision was 0.4% higher and recall was 3.4% higher. Workers expressed little preference between the two variants of helper images. On average, precision and recall with images of the first variant were only 0.5% greater and 4% less than precision and recall of the second variant, respectively. However, including the parent image in the stack decreased both precision and recall by 6% and 4% respectively. (TIF) [file pcbi.1009274.s012.tif]

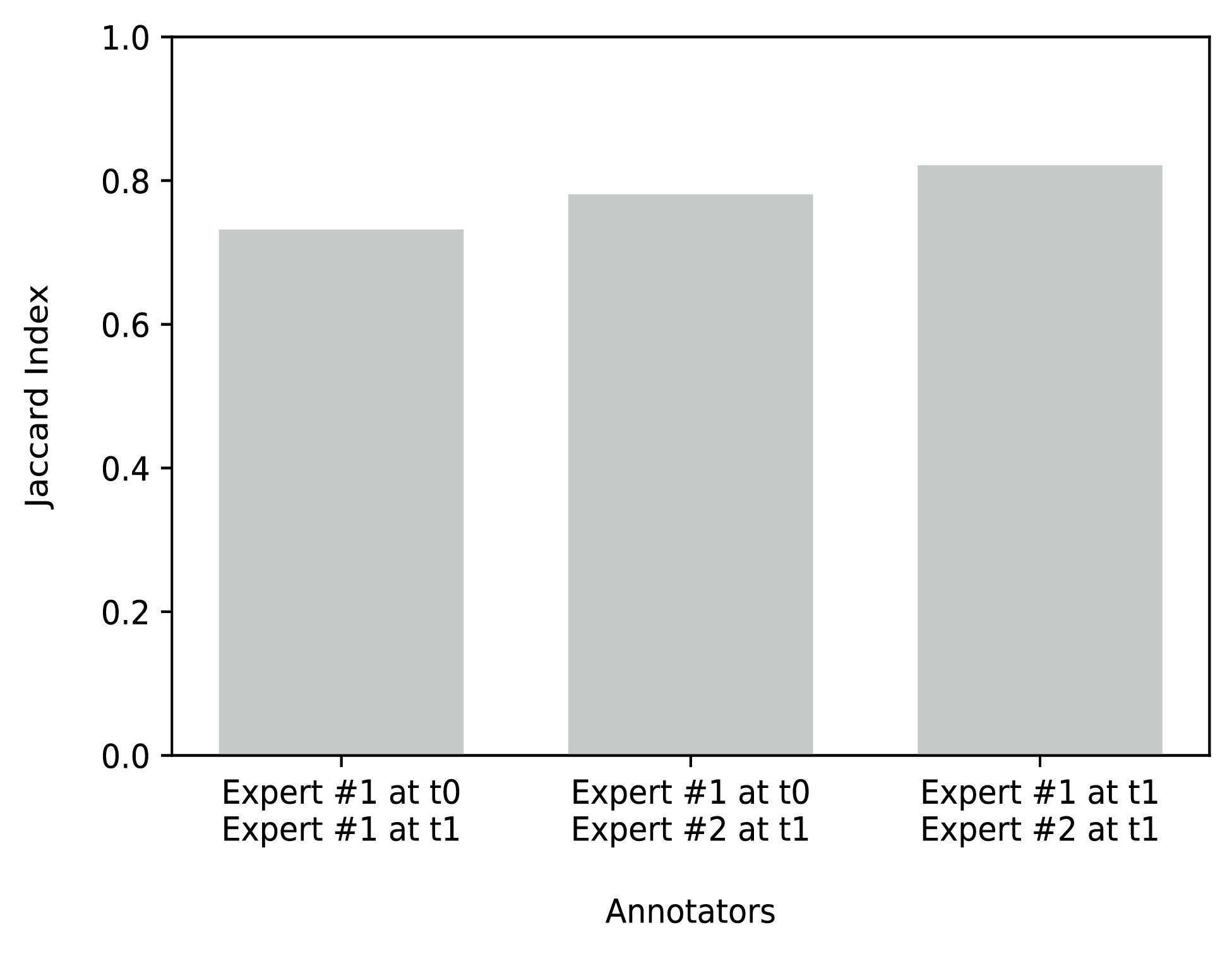

Supplement: S13 Fig — To better contextualize the performance of the consensus annotations, we evaluated the level of concurrence we should expect among experts. The original (t0) expert annotations which have been used as ground truth to evaluate the consensus annotations for the three RCA test images were compared with two new sets of annotations produced for the same data half a year later (t1): one set produced by the same expert (Expert #1) and one set produced by another expert (Expert #2). The Jaccard similarity indices (intersection over union) for Expert #1 at t0 and Expert #1 at t1, Expert #1 at t0 and Expert #2 at t1, and Expert #1 at t1 and Expert #2 at t1 were 73%, 78%, and 82% respectively. (TIF) [file pcbi.1009274.s013.tif]

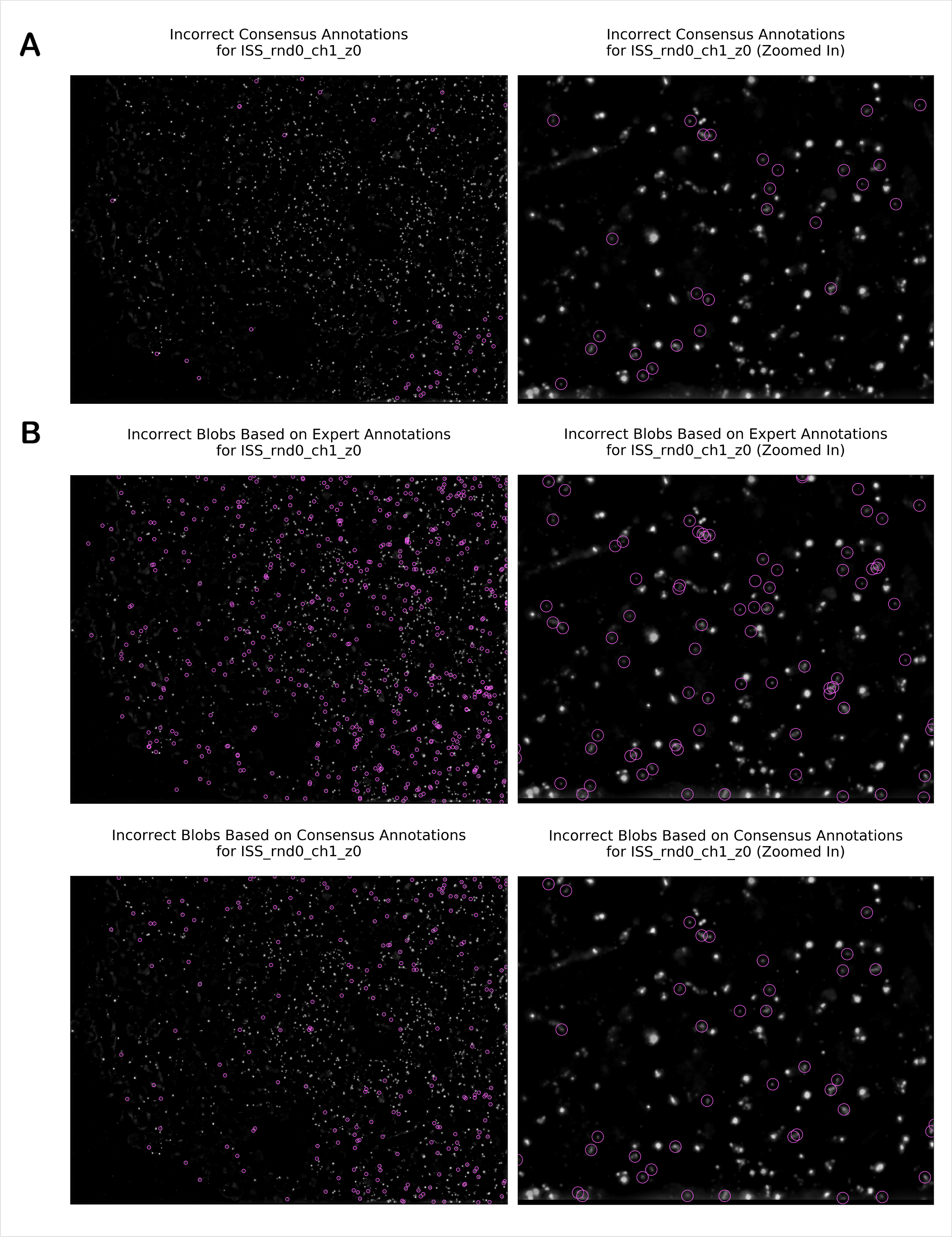

Supplement: S14 Fig — (A) Consensus annotations are more likely to include false positives for specks of debris that experts would ignore. (B) However, for an image from the RCA dataset, the blobs found using scikit-learn’s blob_log() algorithm with parameters based on worker consensus annotations had higher precision (84.8%) than the blobs found using the same algorithm with parameters based on expert annotations (75.1%). (TIF) [file pcbi.1009274.s014.tif]

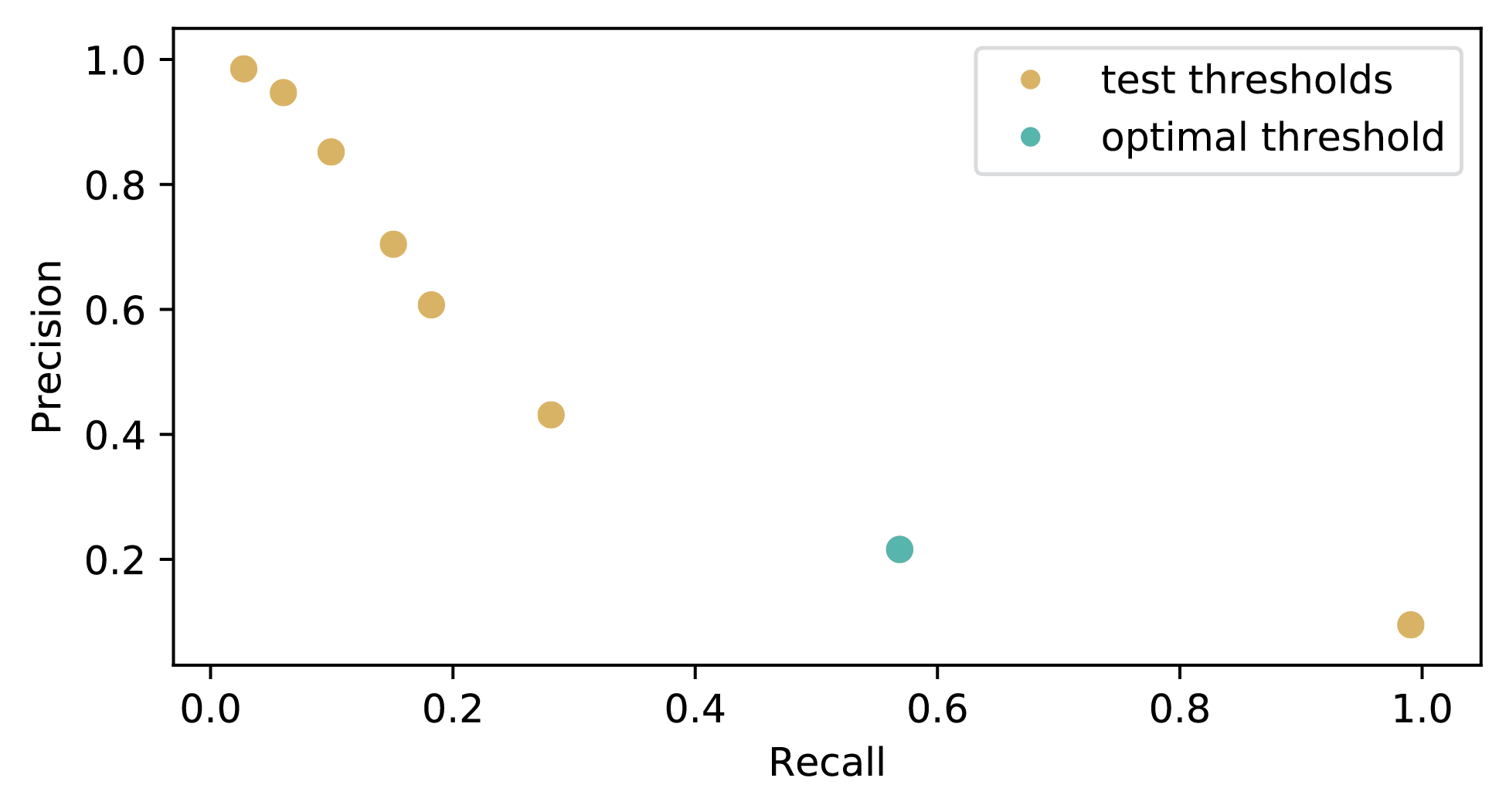

Supplement: S15 Fig — While Starfish’s BlobDetector algorithm detected RCA spots with high precision and recall when parameters were optimized (as demonstrated in Section V, precision and recall for the consensus annotations were 95% and 70%, 92% and 89%, and 81% and 76% for images ISS_rnd0_ch1_z0, ISS_rnd0_ch3_z0, and ISS_rnd1_ch1_z0 respectively), the same algorithm performed poorly with osmFISH (21), failing to find a threshold parameter which yielded a precision*recall score better than 0.1219 (precision = 20.7%, recall = 59.0%). (TIF) [file pcbi.1009274.s015.tif]

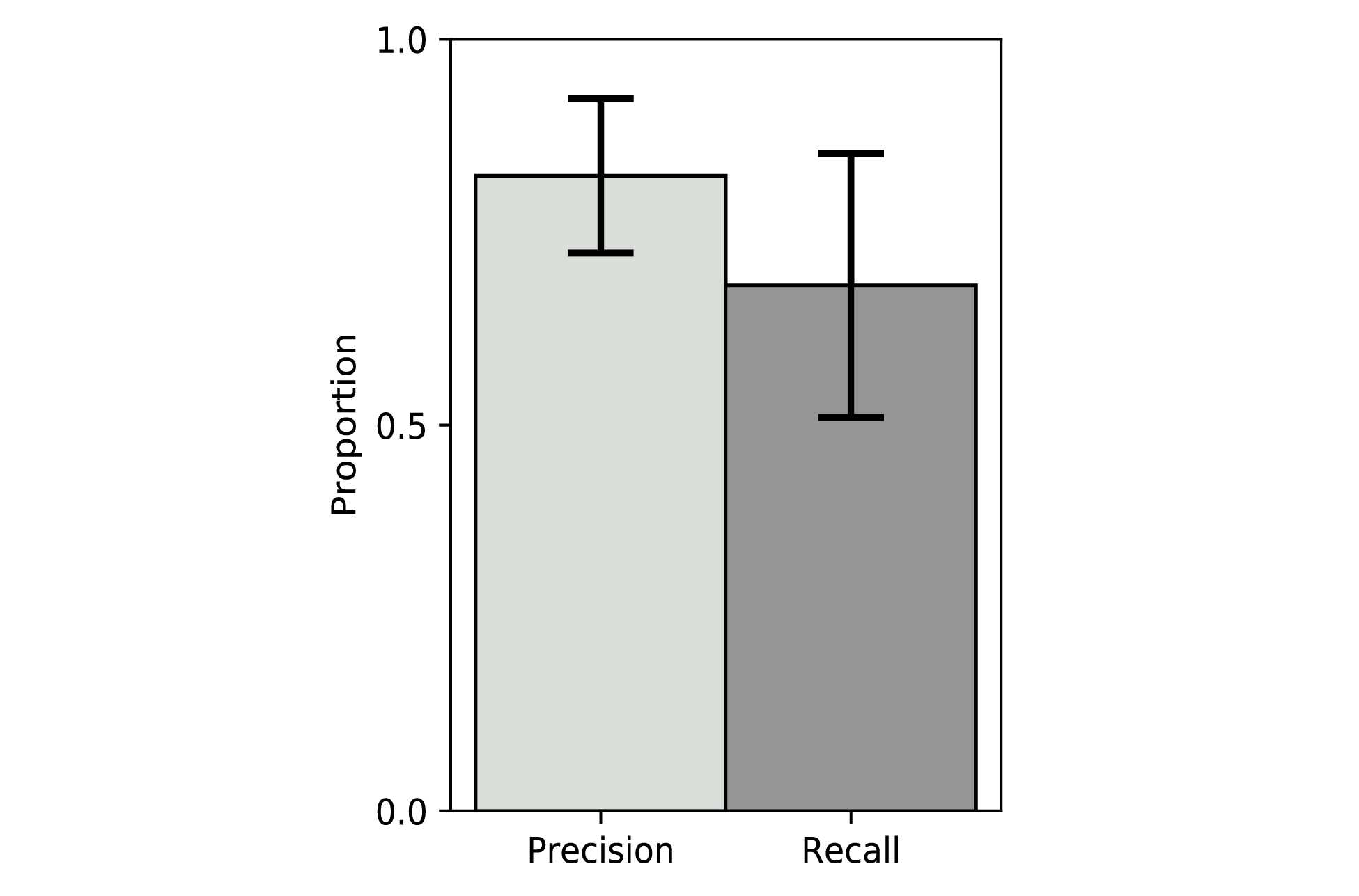

Supplement: S16 Fig — To assess the generalizability of the tuned spot-calling parameters, we ran Starfish’s BlobDetector method using the spot parameters which had been extracted in this vignette on thirteen other images from Starfish’s RCA dataset which had not been annotated by experts. As ground truth, we used consensus annotations for these images. The mean precision was 82% with a standard deviation of 10%, and the mean recall was 68% with a standard deviation of 17%. These results suggest that when a set of spot parameters tuned to a particular channel and field of view for a chemistry are used for other channels and fields of view for the same chemistry, the spots detected are likely to be correct but fewer spots may be detected. (TIF) [file pcbi.1009274.s016.tif]

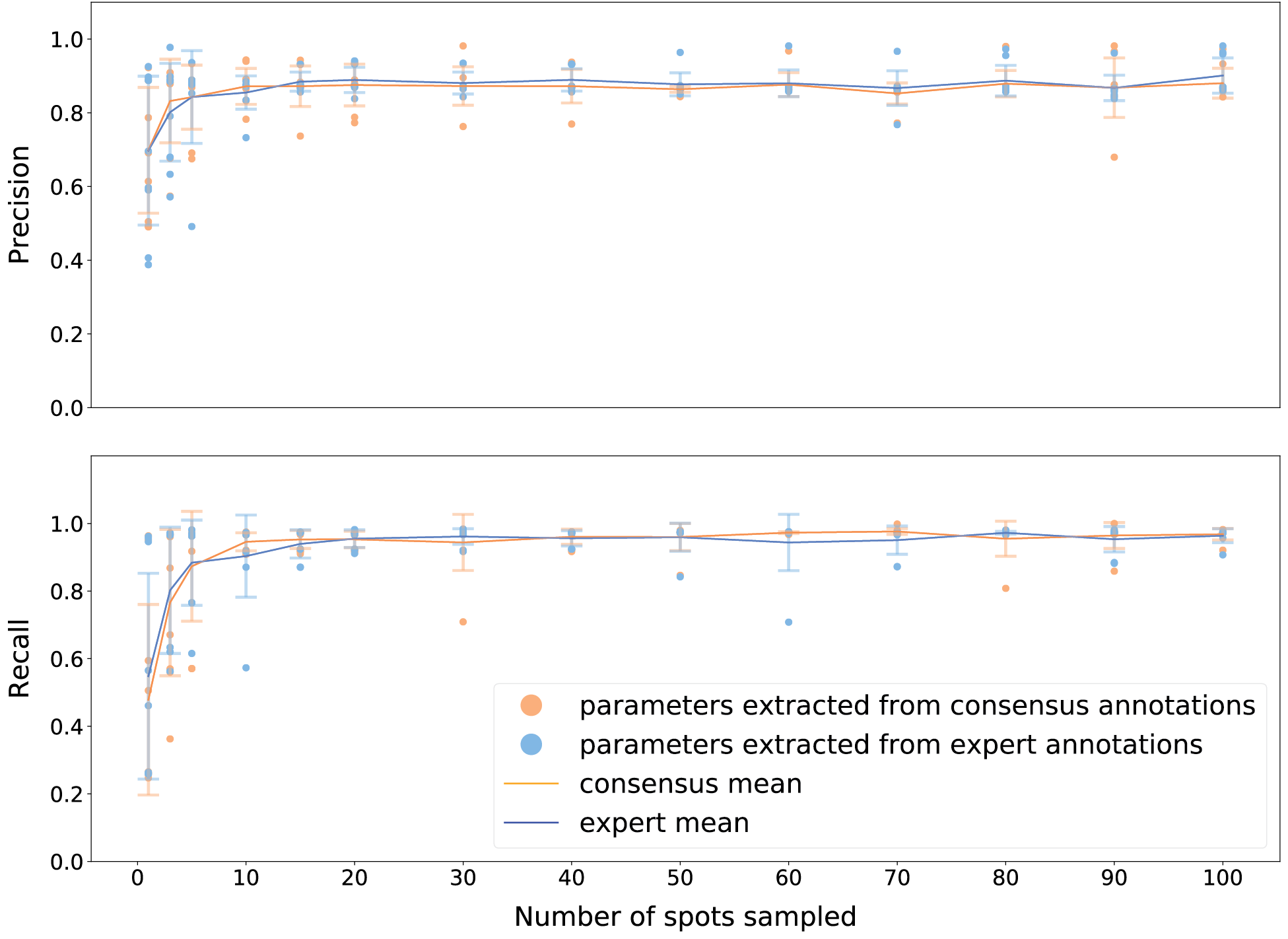

Supplement: S17 Fig — Moreover, when blob detection was executed on ISS_rnd0_ch1_z0, an RCA image from the Starfish dataset that contains 1236 spots, only about 15 or 20 ground truth annotations were needed in order to assure sufficient coverage across the range of spot sizes and intensities and thus get reliable spot size parameters, regardless of whether parameters had been extracted from either expert or consensus ground truth annotations. Above 15 or 20 ground truth annotations, using more sample spots did not significantly improve precision and recall percentage, which leveled off in the high eighties and mid nineties respectively. (TIF) [file pcbi.1009274.s017.tif]
