# Supplementary material for: Validation and tuning of in situ transcriptomics image processing workflows with crowdsourced annotations
Source: PLoS Comput Biol. 2021 Aug 9;17(8):e1009274. doi: 10.1371/journal.pcbi.1009274 (PMC8376178; doi:10.1371/journal.pcbi.1009274)
Supplement: S1 Text — (DOCX) [file pcbi.1009274.s018.docx]

**S1 Text.**

SpotImage is a tool for generating synthetic images with customizable characteristics that simulate real *in situ* transcriptomics images. It facilitates experiments with crowdsourced annotator behavior because ground truth in the synthetic images is perfectly known. The user can assign a biologically realistic background image and control characteristics of the spots including number, size, shape, crowdedness, SNR, and distribution across the image. A few generated images can be found in S7A Fig.

Demo notebook here: <https://github.com/czbiohub/SpotImage/tree/master/demo>**.**
