# Supplementary material for: Validation and tuning of in situ transcriptomics image processing workflows with crowdsourced annotations
Source: PLoS Comput Biol. 2021 Aug 9;17(8):e1009274. doi: 10.1371/journal.pcbi.1009274 (PMC8376178; doi:10.1371/journal.pcbi.1009274)
Supplement: S2 Text — (DOCX) [file pcbi.1009274.s019.docx]

**S2 Text.**

The images which we send to Quanti.us typically contain between 50 and 75 spots each. Using simulated spot images, we found that at least 20 workers are necessary to consistently yield precision and recall greater than 95% for images which contain 75 spots, and that the number of workers required for reliable annotation of an image does not increase dramatically as the number of spots in the image increases (S3 Fig). We used a few extra workers for added insurance because each replicate only costs five cents.
