# Supplementary material for: Validation and tuning of in situ transcriptomics image processing workflows with crowdsourced annotations
Source: PLoS Comput Biol. 2021 Aug 9;17(8):e1009274. doi: 10.1371/journal.pcbi.1009274 (PMC8376178; doi:10.1371/journal.pcbi.1009274)
Supplement: S3 Text — (DOCX) [file pcbi.1009274.s020.docx]

**S3 Text.**

For 1,525 simulated annotation clusters on mouse lung tissue background images annotated by 25 turkers each, thresholding by number of annotations based on assumption of a bimodal distribution resulted in a mean sensitivity of 93.8% with a standard deviation of 17.2% and a mean specificity of 98.6% with a standard deviation of 3.7%.
