# Supplementary material for: Validation and tuning of in situ transcriptomics image processing workflows with crowdsourced annotations
Source: PLoS Comput Biol. 2021 Aug 9;17(8):e1009274. doi: 10.1371/journal.pcbi.1009274 (PMC8376178; doi:10.1371/journal.pcbi.1009274)
Supplement: S4 Text — (DOCX) [file pcbi.1009274.s021.docx]

**S4 Text.**

**Filtering (Main Text Fig 3A)**

Background signal is removed using a Gaussian high-pass filter. Then, the spots are enhanced with a Laplace filter. Sigma values for these filters are chosen manually based on the characteristics of the image. Finally, taking the maximum projection over z mitigates the effects of out of focus z-planes. These filters are implemented in the Starfish python library. The protocol is described in this publication (<https://www.nature.com/articles/s41592-018-0175-z>) and the usage of these filters is demonstrated in this notebook.

**Cropping (Main Text Fig 3B)**

For each image, the Laplacian of Gaussian algorithm is used to execute first-pass blob detection. We use scikit-learn’s implementation of this algorithm, blob_log() (<https://scikit-image.org/docs/dev/api/skimage.feature.html#skimage.feature.blob_log>). The parameters given to blob_log() for spot brightness and size can be extracted from a sample image of the same chemistry with expert annotations by extracting the maximum intensity of the annotated spots and the range of sigma values associated with their Gaussian approximations.

If a sufficiently small (defined by the user) proportion of the detected spots are “crowded,” then the image is deemed usable. That is, for each spot, the distance to its nearest neighbor (NND) is calculated. Since in Quanti.us user clicks leave crosshair marks over marked spots that can obscure neighboring spots, we use NND < [image width * (ratio of crosshair width to image width in Quanti.us)] as the benchmark for “crowded” spots.

If too many spots are crowded, then another level of crops is generated. Each level of cropping happens in three steps. Firstly, clustering is executed on all crowded spots. We use the AffinityPropagation clustering algorithm as implemented by scikit-learn, starting with the preference parameter set to -500 (<https://scikit-learn.org/stable/modules/generated/sklearn.cluster.AffinityPropagation.html>). Depending on the number and distribution of spots, the first clustering attempt might return far more clusters than would be useful. The user can specify the maximum number of clusters that should be returned. If more clusters are found, the preference parameter is adjusted and clustering is reattempted. After five iterations of attempted clustering, the crowded spots are partitioned into five clusters using scikit-learn’s implementation of k-means clustering in 2D (<https://scikit-learn.org/stable/modules/generated/sklearn.cluster.KMeans.html>). Secondly, a bounding box is then drawn around each group of spots, creating child images, and the parent image is blacked out where the child images were. Thirdly, crowdedness is assessed for each child image and another level of crops is generated if necessary. This recursive cropping is implemented in the annotation pipeline/class.
