# Supplementary material for: Validation and tuning of in situ transcriptomics image processing workflows with crowdsourced annotations
Source: PLoS Comput Biol. 2021 Aug 9;17(8):e1009274. doi: 10.1371/journal.pcbi.1009274 (PMC8376178; doi:10.1371/journal.pcbi.1009274)
Supplement: S5 Text — (DOCX) [file pcbi.1009274.s022.docx]

**S5 Text.**

In rolling circle amplification (RCA), circularized padlock probes are amplified continuously with a DNA polymerase, resulting in a single-stranded DNA concatemer with repeating copies of the original padlock probe sequence.[1 ] The product can be detected using fluorescence *in situ* hybridization (FISH) probes, by *in situ* sequencing, via sequencing by ligation, or via sequencing by synthesis. We chose RCA-plus-FISH (referred to as RCA only from now on) for this vignette because RCA images contain a high enough density of signals to demonstrate the utility of the image preparation and cluster QC tools, and because the spots are more highly varied in size than in other chemistries (S9A Fig).

**References**

1. [**Beliveau BJ, Joyce EF, Apostolopoulos N, Yilmaz F, Fonseka CY, McCole RB, et al. Versatile design and synthesis platform for visualizing genomes with Oligopaint FISH probes. Proc Natl Acad Sci U S A. 2012 Dec 26;109(52):21301–6.**](https://www.zotero.org/google-docs/?zuEFED)
