# Supplementary material for: Validation and tuning of in situ transcriptomics image processing workflows with crowdsourced annotations
Source: PLoS Comput Biol. 2021 Aug 9;17(8):e1009274. doi: 10.1371/journal.pcbi.1009274 (PMC8376178; doi:10.1371/journal.pcbi.1009274)
Supplement: S6 Text — (DOCX) [file pcbi.1009274.s023.docx]

**S6 Text.**

**Inputs**

The inputs to the pipeline were one sample image with the RCA chemistry, expert spot location annotations for that image, and three test images without annotations. We began with images downloaded from an *in situ* sequencing (ISS) experiment in the Starfish database ([Reproduce In-situ Sequencing results with Starfish [Internet]. GitHub. [cited 2020 Jun 24]. Available from:](https://www.zotero.org/google-docs/?zuEFED) <https://github.com/spacetx/starfish/blob/master/notebooks/ISS.ipynb>). Using Starfish, we pre-processed (applied filters to) the images as described in Section IV. Of these, we selected one image which qualitatively appeared to be representative of the others, by visual inspection. An expert annotated this image. This image was designated the “sample image,” and the others were designated “test images” (S9A Fig).

**Pipeline**

In the first step, the spots which the expert annotator had annotated in the sample image were analyzed to extract spot detection parameters intaken by Starfish’s BlobDetector method, which implements the Laplacian of Gaussian spot detection approach. This method requires two parameters: sigma_max, and threshold. The sigma_max parameter indicates the maximum size of a spot that can be detected. A sigma_max which is too small results in undetected spots (false negatives). A sigma_max which is too large results in detecting large background blobs, and aberrations in the image, as spots (false positives). Our parameter extraction method designates the largest sigma associated with a spot annotated by the expert as sigma_max (S10A Fig). The threshold parameter indicates the lower bound on the brightness of a detected spot. A threshold that is too large results in missing spots. A threshold that is too small results in detecting background noise, fuzz, and small aberrations as spots. Our parameter extraction method chooses the threshold which optimizes precision times recall when the BlobDetector method is executed on the sample image (S10B Fig). The maximum sigma and optimal intensity threshold found with this parameter extraction are now considered "tuned" parameters. The performance of the BlobDetector method with these parameters can be measured against reliable consensus annotations in other images of the same chemistry. The subsequent steps of the pipeline will get these reliable consensus annotations for each image. These later steps assume that our spot-calling algorithm, the BlobDetector method, with the extracted parameters, provides a sufficiently good first-pass detection to provide a general idea of where the spots are, so that recursive cropping can zoom into those regions.

In the second step, blob detection with the spot parameters found above was executed using the BlobDetector method on each test image. Blob detection provided a general idea of the regions where spots were located (S9B Fig).

In the third step, the coordinates of the spots detected by blob detection were used to automatically subdivide the test images as described in Section IV (S9B Fig). The results of automatic subdivision varied depending on the amount and distribution of crowded spots. The first two test images had crowded spots in most regions where spots were present, so automatic subdivision resulted in the fragmentation of the region where spots were present. The third test image had fewer crowded spots in general and multiple disparate regions were cropped instead. The crops were then sent to Quanti.us to be annotated by 25 workers each. The annotations for each image were then clustered and the clusters were QC’d according to the method described in Section III. The resulting consensus annotations from the individual crops were then reassembled, resulting in consensus annotations for each of the original images (S9C Fig). These consensus annotations are an output of the total pipeline.

In the last step, the performance of the consensus annotations was evaluated based on expert evaluations of the test images.
