# Supplementary material for: Validation and tuning of in situ transcriptomics image processing workflows with crowdsourced annotations
Source: PLoS Comput Biol. 2021 Aug 9;17(8):e1009274. doi: 10.1371/journal.pcbi.1009274 (PMC8376178; doi:10.1371/journal.pcbi.1009274)
Supplement: S7 Text — (DOCX) [file pcbi.1009274.s024.docx]

**S7 Text.**

The original (t_0_) expert annotations which were used as ground truth to evaluate the consensus annotations for the three RCA test images were compared with two new sets of annotations produced for the same data half a year later (t_1_): one set produced by the same expert (Expert #1) and one set produced by another expert (Expert #2). This intra- and inter-expert concurrence gives us an idea of the highest performance that can be expected from crowdsourced annotations.
