# Supplementary material for: Validation and tuning of in situ transcriptomics image processing workflows with crowdsourced annotations
Source: PLoS Comput Biol. 2021 Aug 9;17(8):e1009274. doi: 10.1371/journal.pcbi.1009274 (PMC8376178; doi:10.1371/journal.pcbi.1009274)
Supplement: S8 Text — (DOCX) [file pcbi.1009274.s025.docx]

**S8 Text.**

When testing how well a spot-calling algorithm generalizes to other *in situ* transcriptomics chemistries, ground truth for each chemistry being tested is essential to avoid overestimating the generalizability. For example, while Starfish’s BlobDetector algorithm used on RCA spots produced spot locations good enough for subsequent automatic subdivision of the images, the same algorithm performed poorly with cyclic-ouroboros single molecule fluorescence *in situ* hybridization (osmFISH) [ 1], failing to find a threshold parameter which yielded a precision*recall score better than 0.1219 (precision = 20.7%, recall = 59.0%) (S15 Fig). This might be because the spots in osmFISH images are much lower in contrast than the spots in RCA images, even after filtering. The precision*recall score is useful because there is a tradeoff between precision and recall when the brightness threshold increases – higher precision results in lower recall and vice versa – so optimizing precision*recall is a standardized way to identify the optimum threshold.

**References**

1. [**Lindeberg T. Feature Detection with Automatic Scale Selection. Int J Comput Vis. 1998 Nov 1;30(2):79–116.**](https://www.zotero.org/google-docs/?zuEFED)
