# Supplementary material for: Validation and tuning of in situ transcriptomics image processing workflows with crowdsourced annotations
Source: PLoS Comput Biol. 2021 Aug 9;17(8):e1009274. doi: 10.1371/journal.pcbi.1009274 (PMC8376178; doi:10.1371/journal.pcbi.1009274)
Supplement: S9 Text — (DOCX) [file pcbi.1009274.s026.docx]

**S9 Text.**

We then sought to test whether consensus and expert annotations function similarly well as ground truth for tuning a spot-calling algorithm and to explore the minimum number of ground truth annotations needed to find the spot size parameter. Multiple sets of annotations were sampled from both expert and worker consensus annotations. Spot size parameters were extracted from these sets of annotations, and the intensity threshold parameter was found using these extracted spot size parameters as well as all the ground truth available for the image, by necessity. BlobDetector was run using these extracted parameters. This was repeated ten times for each set of expert and worker consensus annotations. The results are shown in S17 Fig.

Across different numbers of spots, precision and recall were respectively just 0.67% and 1.33% different when expert annotations and consensus annotations were used as ground truth, and training behavior was very similar between the two types of annotations (S17 Fig). Moreover, when blob detection was executed on RCA image ISS_rnd0_ch1_z0, which contains 1236 spots, 15 ground truth annotations were enough to get 99.1% and 98.1% of the maximum precision performance when the annotations were produced by experts and worker consensus, respectively. The same number of annotations was enough for 97.6% and 96.6% of the maximum recall performance with annotations produced by experts and worker consensus, respectively. These results suggest that for the RCA chemistry, about 15 ground truth annotations were needed in order to assure sufficient coverage across the range of spot sizes and thus get reliable spot size parameters. Above 15 ground truth annotations, using more sample spots did not significantly improve precision and recall, which leveled off in the high eighties and mid nineties percent, respectively. These results suggest that while consensus annotations are useful when large amounts of ground truth are needed to check or validate the performance of spot-calling algorithms, a few dozen expert annotations alone may be sufficient to begin to tune a spot-calling algorithm such as Starfish’s BlobDetector.

While a few dozen expert annotations alone may be sufficient for tuning a spot detector, as demonstrated in S17 Fig, consensus annotations are critical to provide the large quantity of ground truth required to check or validate the performance of spot-calling algorithms, since for validation ground truth must be provided for every spot on every image in a given dataset.
