# Supplementary material for: Validation and tuning of in situ transcriptomics image processing workflows with crowdsourced annotations
Source: PLoS Comput Biol. 2021 Aug 9;17(8):e1009274. doi: 10.1371/journal.pcbi.1009274 (PMC8376178; doi:10.1371/journal.pcbi.1009274)
Supplement: S10 Text — (DOCX) [file pcbi.1009274.s027.docx]

**S10 Text.**

**Balancing the tradeoff between crop detail and crowdsourcing cost:** If a cheap experiment yields a very large dataset with many images, a user may be less concerned with maximizing data extracted from each image, but if each image costs more to produce, the researcher might wish to be more detailed with cropping. For example, assume an experiment yields images with 1000 spots each (as in RCA test image 1). Assume 25 replicates are desired and each replicate costs five cents. This results in a cost of $1.25 per image (without cropping), but we know that with 1000 spots, the fraction of spots annotated will be low. From Fig 4C we see that when images have 200 spots each, it is more reasonable to expect full or almost full coverage with 25 replicates. Additionally, Fig 5C (smFISH cropping demo) shows that automatic subdivision of the images can vastly improve recall – that is, the amount of data retrieved from an image. Consequently, dividing the original image into 5 sub-images (each with about 200 spots) before annotating will greatly improve recall at a 5x increase in cost.
