# Supplementary material for: Validation and tuning of in situ transcriptomics image processing workflows with crowdsourced annotations
Source: PLoS Comput Biol. 2021 Aug 9;17(8):e1009274. doi: 10.1371/journal.pcbi.1009274 (PMC8376178; doi:10.1371/journal.pcbi.1009274)
Supplement: Attachment — Submitted filename: INSTA Resubmission Point-by-Point.docx [file pcbi.1009274.s028.docx]

**Point-by-point response to Reviewer comments on PCOMPBIOL-D-20-01316.**

Reviewers’ comments are *italicized*; new/revised material is in blue; author responses are presented directly after each comment.

**R#1 preface comments:** “...*the proposed pipeline is rigorously developed, the experiments are well designed, and the manuscript is generally well structured and written. From a technical perspective, the overall approach is sound…”*

We appreciate that Reviewer 1 acknowledges that the manuscript seeks to address analysis challenges facing the field of *in situ* transcriptomics. In addition, we are grateful for the reviewer’s incisive comments and helpful suggestions. We aimed to address their concerns as detailed below.

**R#1 C1:** *What I miss in the paper is, first and foremost, a discussion on how this falls in a wider in-situ sequencing transcriptomics workflow.*

We thank the reviewer for pointing out the need to clarify this. We accordingly added this paragraph before the last paragraph in the introduction (page 6) and a supplementary figure (S1 Figure) to illustrate.

A user would incorporate INSTA into a wider in-situ sequencing transcriptomics workflow as follows (S1 Figure): (1) Run test experiment with the chosen chemistry. (2) Check SNR and spot density (in the Results section, we provide guidance on the characteristics which make an image suitable for INSTA). (3) Expert annotates some images. (4) Use these annotations to try tuning a spot-finding algorithm. (5) Decide if crowd-sourcing annotations are necessary. (6) If so, consider running more experiments to get more images. (7) Use INSTA to prepare the images for crowdsourcing the annotations. (8) Send out images for annotations. (9) Receive annotations and process them with INSTA. (10) Tune and benchmark a spot-finding algorithm using the consensus annotations.

***R#1 C2:*** *Moreover, given that this can be applied in a wide variety of biological and experimental settings, I would feel exploration of the following questions should also be addressed:*

A. *What is the impact of different types of tissue. For example, do crowdsourced annotations fare equally well in complex, heterogeneous tissue with varying levels of pathology?*
B. *Different genes will have different RNA location patterns throughout a cell. There are RNA species predominantly nuclear vs. cytoplasmic. E.g., different genes will have a different proclivity to clump. Does this have any impact?*
C. *There are many in-situ sequencing technologies available. How well does crowdsourcing apply to the different technologies?*
D. *Do different imaging settings have an impact (e.g., different fluorescence channels)?*

We thank the reviewer for pointing out that these questions should be addressed. We accordingly reframed the discussion of the effect of image properties on performance in the context of sample and chemistry choices and to better show how this toolkit falls in a wider *in situ* sequencing transcriptomics workflow.

In particular, we did the following things:

(1) We inserted the following paragraphs at the beginning of the section “Worker performance is limited by spot crowding, visibility, and quantity” to help the reader understand how the image properties affect performance. These additions describe the impact of different (a) types of tissue, (b) spatial distributions of genes, (c) *in situ* technologies, and (d) imaging settings.

Given the large number of *in situ* transcriptomics technologies and even larger number of tissues to analyze, we sought to understand the fundamental properties of the images required for high quality crowd-sourced annotations. In particular, we aimed to characterize worker performance as a function of signal-to-noise ratio (SNR) and the distances between spots. We hypothesized that these two properties of *in situ* transcriptomics images would be fundamental determinants of annotation quality.

Many factors in sample preparation and imaging affect the SNR of spots in *in situ* transcriptomics images. For example, some tissues exhibit higher autofluorescence owing to the composition of the cells and extracellular matrix.[(32,33)](https://www.zotero.org/google-docs/?8vxZIl) Light scattering due to tissue composition further degrades SNR, with shorter wavelengths generally more degraded than longer wavelengths.[(34)](https://www.zotero.org/google-docs/?FokamU) Understanding how the protocol chemistry and tissue properties affect performance can help guide the sample preparation optimization process. For example, optimizing sample preparation via tissue clearing [(35)](https://www.zotero.org/google-docs/?opA7iv) and fixative selection [(36)](https://www.zotero.org/google-docs/?TdGh9n) can improve SNR and contrast.

The ability to resolve adjacent spots depends on both their physical distances and the magnification and microscope quality used to image them. The physical distances between mRNA molecules in the cell vary both by their abundance and localization. Dense transcripts can be imaged with higher magnification, which can improve resolution at the cost of imaging speed. Alternatively, expansion microscopy can be used to improve the spatial resolution of transcripts.[(37,38)](https://www.zotero.org/google-docs/?y7mKg0) Anticipating that dense transcripts could present a challenge for workers to annotate, we developed quality control methods to identify and declump annotations from dense spots, and created the automatic image subdivision system.

We crowdsource annotations using Quanti.us (30) and perform clustering and quality control on the annotations to arrive at consensus coordinates for spot locations…

**References added:**

[32.	Pavlova I, Williams M, El-Naggar A, Richards-Kortum R, Gillenwater A. Understanding the Biological Basis of Autofluorescence Imaging for Oral Cancer Detection: High-Resolution Fluorescence Microscopy in Viable Tissue. Clin Cancer Res. 2008 Apr 15;14(8):2396–404.](https://www.zotero.org/google-docs/?zuEFED)

[33.	Monici M. Cell and tissue autofluorescence research and diagnostic applications. In: Biotechnology Annual Review [Internet]. Elsevier; 2005 [cited 2021 Mar 4]. p. 227–56. Available from: https://www.sciencedirect.com/science/article/pii/S1387265605110072](https://www.zotero.org/google-docs/?zuEFED)

[34.	Helmchen F, Denk W. Deep tissue two-photon microscopy. Nat Methods. 2005 Dec;2(12):932–40.](https://www.zotero.org/google-docs/?zuEFED)

[35.	Moffitt JR, Hao J, Bambah-Mukku D, Lu T, Dulac C, Zhuang X. High-performance multiplexed fluorescence in situ hybridization in culture and tissue with matrix imprinting and clearing. Proc Natl Acad Sci. 2016 Dec 13;113(50):14456–61.](https://www.zotero.org/google-docs/?zuEFED)

[36.	Lee K, Choi S, Yang C, Wu H-C, Yu J. Autofluorescence generation and elimination: a lesson from glutaraldehyde. Chem Commun. 2013 Mar 14;49(29):3028–30.](https://www.zotero.org/google-docs/?zuEFED)

[37.	Wang G, Moffitt JR, Zhuang X. Multiplexed imaging of high-density libraries of RNAs with MERFISH and expansion microscopy. Sci Rep. 2018 Mar 19;8(1):4847.](https://www.zotero.org/google-docs/?zuEFED)

[38.	Alon S, Goodwin DR, Sinha A, Wassie AT, Chen F, Daugharthy ER, et al. Expansion sequencing: Spatially precise in situ transcriptomics in intact biological systems. Science [Internet]. 2021 Jan 29 [cited 2021 Mar 4];371(6528). Available from: https://science.sciencemag.org/content/371/6528/eaax2656](https://www.zotero.org/google-docs/?zuEFED)

(2) We also broke up the large paragraph starting on page 13 reporting our characterization of the effect of SNR, NND, spot size on annotation performance and put each finding into context, linking the parameter values we explored to key experimental aspects such as tissue types, *in situ* technologies, and imaging settings (addressing questions A, C, and D from R#1 C2 above).

We observed some limits on the utility of QC. When spot visibility was poor, nearest neighbor distance was very small, or an image had too many spots, the quality of the raw annotations was very low, so QC was unable to improve precision and recall to acceptable levels.

Using the NND-varying and SNR-varying features of our SpotImage tool, we tested the limits of spot visibility and crowdedness that workers can accurately annotate. In an experiment with synthetic images of mean SNR = 3, 5, 7, 9, and 11, and spot size = 0.5, 1.0, and 1.75 pixels (S7a Figure), small spot sizes required larger mean SNR to achieve recall greater than 50%. For example, when the spot size (sigma of a gaussian fit to the intensity) was half a pixel (about 5 microns), recall was zero until SNR > 9 (S7b Figure). At lower SNR values, even spots with large nearest neighbor distances tended to be missed, and as spot SNR increased from 6.5 to 11.5, the mean NND of undetected spots decreased from 14 pixels to about 3 pixels (S7c Figure). While the size of cellular structures in pixels will be a function of the imaging system and chemistry used, note that the cells in the smFISH image used for Figure 3c are 250 to 300 pixels wide, and 130 to 180 pixels tall. A typical nucleus from an RCA image (e.g. idr0071-feldman-crisprko/experimentE/10X_A11_Tile-0.aligned from Feldman et al. 2018(41)) is approximately 8-12 pixels in diameter.

We observed that the radius of the symbol that Quanti.us uses to mark spots identified by workers limits the minimum nearest neighbor distance between spots which we can reasonably expect workers to discern to 4% of the image’s width. When spots are closer than this distance, the mark on one spot obscures neighboring spots. We tested inverting the images (dark spots on a light background) before submitting them to Quanti.us to see if this would improve worker performance. A linear regression between recall with inversion and recall without inversion resulted in a slope of 1.004, with a Pearson’s correlation coefficient of *r* = 0.985 (S6d Figure), making it clear that inversion is not helpful.

**References added:**

41. [Feldman D, Singh A, Schmid-Burgk JL, Mezger A, Garrity AJ, Carlson RJ, et al. Pooled optical screens in human cells. bioRxiv. 2018 Aug 2;383943.](https://www.zotero.org/google-docs/?zuEFED)

(3) We also added clarification on page 15 to directly help the reader assess if images are suitable for INSTA. This revision provides further actionable guidance on selecting experiment conditions that yield good spot-calling performance, especially with regards to different tissue types, *in situ* technologies, and imaging settings (addressing questions A, C, and D from R#1 C2 above).

These effects of spot visibility, crowding, and quantity on worker performance are intuitive, but these results provide quantitative metrics to understand worker performance as a function of image quality, and objective guidelines for image pre-processing that allow us to optimize the performance for a given image dataset. We found that for best performance the spot signal-to-noise ratio must be at least 10 and the mean nearest neighbor center-to-center spot distance must be between 12 and 15 pixels. The performance characterization in this section provides targets for the SNR and density of spots that can aid in guiding the optimization of in situ technologies to new samples. In the next section, we present the methods we developed to prepare images so that workers are more likely to annotate them reliably.

(4) Finally, we provided additional context on page 14 for the number of spots the workers can annotate. This shows the impact of different types of tissue and *in situ* technologies (addressing questions A and C from R#1 C2 above).

We also sought to understand the total number of spots that workers can annotate in one image. In an experiment with SNR = 3, 5, 7 and number of spots per image between 50 and 225, the number of clicks per worker per image increased as the number of spots in the image increased, until it leveled off at around 120 on average, suggesting that 120 was the upper bound on the total number of spots workers were willing to click for the payment offered in these experiments ($0.05 per image) (S8a Figure). For example, in an assay detecting 31 genes in fresh-frozen breast cancer tissue, Ke et al report an average of 25 spots per cell (42), which means that a worker can reliably annotate about 4.8 cells per image. If the average number of spots per cell for one gene is instead 16 [Figure 2b in (43), (44)], the same worker would be able to annotate 7.9 cells per image. As the number of spots per image increased beyond 50, the fraction of spots that workers were willing to click decreased. On average, workers annotated almost all spots for images with 50 spots but only about 60% of all spots for images with 200 spots (S8b Figure). However, even though each worker annotated a smaller fraction of the spots as the number of spots in the image increased, most spots were still getting annotations from at least half the workers (S8c Figure). In other words, some workers detected spots which other workers missed.

**References added:**

[42.	Ke R, Mignardi M, Pacureanu A, Svedlund J, Botling J, Wählby C, et al. In situ sequencing for RNA analysis in preserved tissue and cells. Nat Methods. 2013 Sep;10(9):857–60.](https://www.zotero.org/google-docs/?zuEFED)

[43.	Schwanhäusser B, Busse D, Li N, Dittmar G, Schuchhardt J, Wolf J, et al. Global quantification of mammalian gene expression control. Nature. 2011 May;473(7347):337–42.](https://www.zotero.org/google-docs/?zuEFED)

[44.	Lubeck E, Coskun AF, Zhiyentayev T, Ahmad M, Cai L. Single-cell in situ RNA profiling by sequential hybridization. Nat Methods. 2014 Apr;11(4):360–1.](https://www.zotero.org/google-docs/?zuEFED)

**R#1 C3:** *As the authors indicate, results from either crowdsourced or expert annotated images will be used to train and validate spot detectors. As this paper discusses how expert annotation compares to crowdsourced annotation, I would expect - apart from a statistical quantification of how precise crowdsourced annotation is - an investigation of the impact on the training of spot detectors - i.e., - are there any implicit biases between experts and non-experts that impact downstream training of the model?*

We thank the reviewer for pointing out the need to show whether there are any “implicit biases” between experts and non-experts that impact downstream training of the model. Towards this, we explored if there is a bias in the type of error experts vs. annotators make. We have added the following discussion to the Results section and illustrated the findings with S14 Figure:

Page 18:

We sought to evaluate how well the resulting crowdsourced annotations agreed with expert annotations. Precision and recall for the consensus annotations, based on an expert’s evaluations of the test images, were 95% and 70%, 92% and 89%, and 81% and 76% for images ISS_rnd0_ch1_z0, ISS_rnd0_ch3_z0, and ISS_rnd1_ch1_z0 respectively (Figure 4). The Jaccard similarity indices (intersection over union) between the consensus annotations and the expert annotations were 71%, 85%, and 68%, respectively. We then sought to understand how well the consensus annotations and the expert annotations agreed with each other by evaluating the level of concurrence between different experts and between the same expert annotating the same image twice six months apart (at times t_0_ and t_1_= t_0_ + 6 mos.)(S7 Figure). The Jaccard indices (intersection over union) for Expert #1 at t_0_ and Expert #1 at t_1_, Expert #1 at t_0_ and Expert #2 at t_1_, and Expert #1 at t_1_ and Expert #2 at t_1_ were 73%, 78%, and 82% respectively (S13 Figure). Thus, the agreement we saw between the consensus and expert annotations for the RCA images are in the same range as the intra- and inter-expert Jaccard indices. We also observed that consensus annotations are more likely to include false positives for specks of debris that experts would ignore (S14a Figure). These specks are smaller and fainter than the spots an expert would annotate. However, this common error mode in the consensus annotations does not necessarily hamper the performance of a spot detector trained on consensus annotations. For image ISS_rnd0_ch1_z0, the blobs found using scikit-learn’s blob_log() algorithm with parameters based on worker consensus annotations actually had higher precision and recall (84.8% and 73.1%) than the blobs found using scikit-learn’s blob_log() algorithm with parameters based on expert annotations (75.1% and 72.8%) (S14b Figure). While recalls differ by only 0.4%, precision is significantly lower (12.1% difference) for blobs found based on expert consensus because the expert identified spots with a larger range of sizes and intensities than the workers. Consequently, parameter tuning that took these edge cases into consideration caused blob_log() to capture more false positives and more spots overall (2019 vs. 1388).

**R#1 C4:** *Moreover, it would also be relevant that the authors design and provide a framework to integrate both consensus annotations and expert annotations and investigate how such fused annotations could improve the results. For example, at least for the starfish database, they could report the combination of both consensus annotations and expert annotations as grand truth; then, the cross posting results could also be incorporated into the current version of Figure 5.*

We thank the reviewer for identifying the need to demonstrate expert and consensus annotations being used together to tune a spot-calling algorithm. To do this, we performed this experiment:

1. Consolidate a master ground-truth dataset containing image(s) with consensus annotations and other image(s) with expert annotations.
2. Use that combined ground-truth dataset to tune the LMP spot detection algorithm (as in Figure 5) and get precision and recall of detection with various values for the stringency parameter. 3.
3. Add that result to Figure 5. Figure 5 will now have results from consensus, expert, and consensus + expert.

We describe this experiment in the **Results** section on page 21.

Usage of consensus annotations and expert annotations as ground truth to tune the LMP (starfish’s LocalMaxPeakfinder) stringency parameter, which controls the intensity cutoff to detect a peak, resulted in similar precision vs. stringency trends, as well as recall vs. stringency trends (Figure 5). The optimal stringency parameter found using consensus annotations from crowdsourced workers alone as ground truth resulted in a lower precision and slightly higher recall (89.4% and 95.4% respectively, compared with 94.3% and 94.8% from using expert annotations alone as ground truth – 5.3% and 0.63% difference respectively). These results reflect the fact that some of the spots annotated by the worker consensus were not annotated by the more conservative expert.

The optimal stringency parameter found using consensus annotations from crowdsourced worker annotations pooled together with the expert’s annotations resulted in precision and recall almost equivalent to the results using consensus annotations from crowdsourced workers alone (89.5% and 95.3% – 0.1% percent difference for both). This result likely reflects the fact that the number of expert annotations is typically much smaller than that of worker annotations, so their effect on the pool is minimal. Nevertheless, combining crowdsourced annotations with expert annotations provides a way to extend the amount of ground truth available.

The updated Figure 5 is in the updated folder of figures.

Link to the new Figure 5 notebook:

<https://github.com/czbiohub/instapaper/blob/resubmission/figures/MT/vignette_notebooks_and_figs/RCA/MT_Fig_5_sensitivity_lmp_0_1.ipynb>

**R#1 C5:** *Figure 1 & Figure 5: Please provide better titles and more descriptive legends.*

We thank the reviewer for pointing out this need.

Since our Figure 1 does not have a legend, we have made the title/caption more descriptive:

**Figure 1. INSTA (IN situ Sequencing and Transcriptomics Annotation) is a pipeline for tuning and validating spot detection methods using crowdsourced annotations.** The steps for optimizing the spot detection parameters specific to a given chemistry are as follows. Row 1: A sample image from that chemistry is filtered and annotated by an expert. From this small amount of expert input, the tool learns what a spot in this chemistry should look like and passes these parameters to a spot detector. Row 2: The images that will receive consensus annotations are individually pre-processed. The spot detector uses the learned parameters to do rough, first-pass spot-calling. A function then detects the crowded regions and recursively zooms in to them until the sub-images are sufficiently uncrowded that a human worker should easily be able to click on all the spots without getting fatigued. Then, all the sub-images and parent images are sent to [Quanti.us](http://quanti.us/), which is a system for crowdsourced annotation through Amazon’s Mechanical Turk platform. Crowdsourced workers annotate each crop. Row 3: Quality control is performed based on characteristics of the spots and clusters to produce consensus annotations, which are then reassembled to produce an original image that has been annotated with high precision and recall. Row 4: These annotations can be used to tune and train other spot detectors. They can also be used to quantify the performance of the spot detector that this run of the pipeline attempted to optimize.

We have updated our Figure 5 legend and caption in light of our update to the experiment it describes. The caption is as follows:

***Figure 5.*** *The optimal “stringency” parameter for starfish’s LocalMaxPeakfinder (LMP) with Rolling Circle Amplification (RCA) images from the starfish database resulted in lower precision and slightly higher recall (89.4% and 95.4% respectively) when consensus annotations were used as ground truth for parameter tuning, compared with 94.3% and 94.8% precision and recall which were achieved when expert annotations were used as ground truth for parameter tuning. The optimal stringency parameter found using consensus annotations from crowdsourced worker annotations pooled together with the expert’s annotations resulted in precision and recall almost equivalent to the results using consensus annotations from crowdsourced workers alone (89.5% and 95.3% – 0.1% percent difference for both).*

**R#1 C6:** *Figure 2b & Suppl. Figure 1, 3, and 5: Please provide higher resolution and/or separate images for the raw signal & the annotations. It is not easy to interpret. In Suppl. Figure 1, the colors are difficult to distinguish.*

These higher resolution, separate, and updated images are in the updated folder of figures.

**R#1 C7:** *Suppl. Figure 6: Do the authors not mean images with a spot SNR running from 1 to 11?*

We thank the reviewer for pointing out this typo in the caption for S7 Figure. We do mean images with a spot SNR ranging from 1 to 11. We have made this correction.

***S7 Figure. Workers are sensitive to spot visibility (signal to noise ratio) and crowdedness (nearest neighbor distance). (A)*** *This experiment used synthetic images with spot SNR ranging from 1 to 11; spot size = 0.5, 1.0, and 1.75; plus inverted and not inverted. A subset of these images are shown here.* ***(B)*** *In an experiment using these images, small spot sizes required larger mean SNR to achieve good recall.* ***(C)*** *At lower SNR values, even spots with large nearest neighbor distances tended to be missed, and as spot SNR increased, the median NND of undetected spots decreased.* ***(D)*** *A linear regression between recall with inversion and recall without inversion resulted in a slope of 1.004 with Pearson’s correlation coefficient of r = 0.985. In other words, there was insufficient evidence that inverting the images improved worker performance.*

**R#2 preface comments:** “...*Overall, I really like this manuscript. Automated processing of spatial transcriptomic data is non-trivial and the nature of the computational task can make it challenging to rely on “out of the box” solutions for spot detection, especially between imaging platforms and/or in situ labeling technologies. Accordingly, deep learning approaches often are the most powerful options for this task. The toolkit introduced here is timely and packed with useful features for preparing imaging data for manual annotation—be it by crowdsourced workers or in-house experts—in order to generate “ground truth” datasets to train the learning models. As crowdsourced annotation is likely to become more and more common for this task, I expect INSTA to be a valuable resource to the growing imaging transcriptomics community.*

*With regards to the manuscript itself, I found it clear and well-written. The figures are clear and easy to follow. The GitHub pages that support INSTA, SpotImage, and the manuscript and well-organized, well-documented and contain useful demos and other tidbits of information to help potential users get going. The data analysis results are well-supported by the approaches taken. I support publication in PLoS Computational Biology, provided the following minor points are addressed...”*

We are thrilled that the reviewer supports publication in PLoS Computational Biology. We aimed to address their helpful feedback as detailed below.

**R#2 C1**: *It would help to make it clearer in the main text how many images and spots are being considered when generating the summary statistics (precision, recall, etc.)*

We thank the reviewer for pointing out this need and have made the following changes:

MT Page 11:

In an experiment with nine synthetic images (with 50, 100, and 150 spots and mean SNR of 5, 10, and 15), this QC step yielded 100%, 100%, and 100% precision (40%, 13%, and 7% increase compared with the precision obtained without thresholding clusters by number of annotations) and 51%, 78%, and 99% recall (19%, 12%, and 0% decrease) for images with mean SNR = 5, 10, and 15 respectively. For each SNR, the reported precision and recall value is the average across the three images with different numbers of spots (S5a Figure).

MT Page 13, Figure 2 caption:

***Figure 2. QC, including cluster size thresholding and declumping, improves precision, sometimes at the expense of recall, for images with lower SNR values. (A)*** *Clusters with fewer workers tend to be incorrect. Sort clusters by number of unique workers annotating them.* *The fraction of workers who contribute once can predict whether a cluster is clumpy (it corresponds to multiple image spots that are close together).* ***(B)*** *Sort clusters by fraction of unique workers contributing. Isolate and declump the clusters where many workers contribute more than once, as the inset demonstrates using real data.* (*Inset – orange circle: original centroid, green and purple circles: new centroids found by declumping, green and purple dots: worker annotations assigned to new centroids by declumping, stars: actual spot locations.) Clumps are declumped using 2D k-means. For more examples of declumped clusters, see S1 Figure.* ***(C)*** *Thresholding clusters by the number of annotations in the cluster and by the fraction of unique workers who contribute multiple times to the cluster improved recall by 17%, while decreasing precision by 11% on average, in an experiment with nine images with 50, 100, and 150 spots; mean SNR = 5, 10, and 15; and average nearest neighbor distance (NND)* ≈ *11.5, 15, and 20.5.*

MT Page 16, Figure 3 caption:

***Figure 3. Images are filtered and subdivided so that they are easier for workers to annotate. (A)*** *The raw image (which contains 268 spots) is pre-processed with a gaussian high pass filter, a Laplace filter, and a maximum intensity projection over z.* ***(B)*** *Crowded spots detected and bound. Rough, first-pass spot-calling enables the detection of crowded spots and subsequent subdivision.* ***(C)*** *True positive = consensus in concordance with expert annotation, false positive = consensus not in concordance with expert annotation, and false negative = no consensus found for an expert annotation. The distance between a correct consensus annotation and the nearest expert annotation is no more than the user-defined correctness threshold. The distance between a detected expert annotation and the nearest consensus annotation is also no more than the user-defined correctness threshold.* ***(D)*** *Applying cropping resulted in precision and recall of 97% and 87%, improvements of 50% and 64%, respectively, over the un-cropped image.*

MT Page 17:

For RCA image ISS_rnd1_ch1_z0, which contains 287 spots, the inclusion of helper images on average increased precision by 14% (95% with helper images and 81% without) and decreased recall by 16% (59% with helper images and 76% without) (S12 Figure). When the spots in the helper image were circled, precision was 0.4% higher and recall was 3.4% higher. Workers expressed little preference between the two variants of helper images. On average, precision and recall with images of the first variant were only 0.5% greater and 4% less than precision and recall of the second variant, respectively. However, including the parent image in the stack, which workers would also annotate, decreased both precision and recall by 6% and 4% respectively.

SI Page 12, S12 Figure caption:

***S12 Figure. Inclusion of helper images improves precision at the expense of recall.*** *For RCA image ISS_rnd1_ch1_z0, which contains 287 spots, the inclusion of helper images on average increased precision by 14% (95% with helper images and 81% without) and decreased recall by 16% (59% with helper images and 76% without). When the spots in the helper image were circled, precision was 0.4% higher and recall was 3.4% higher. Workers expressed little preference between the two variants of helper images. On average, precision and recall with images of the first variant were only 0.5% greater and 4% less than precision and recall of the second variant, respectively. However, including the parent image in the stack decreased both precision and recall by 6% and 4% respectively.*

MT Page 18

We sought to evaluate how well the resulting crowdsourced annotations agreed with expert annotations. Precision and recall for the consensus annotations, based on an expert’s evaluations of the test images, were 95% and 70%, 92% and 89%, and 81% and 76% for images ISS_rnd0_ch1_z0, ISS_rnd0_ch3_z0, and ISS_rnd1_ch1_z0, which contain 1236, 416, and 287 spots respectively (Figure 4).

MT Page 20, Figure 4 caption

***Figure 4. The* in situ *transcriptomics annotation pipeline was tested using RCA (Rolling Circle Amplification) images from an* in situ *sequencing (ISS) experiment in the starfish database.*** *Worker consensus annotations for RCA test image ISS_rnd0_ch3_z0, which contains 416 spots, achieved 92% precision and 89% recall based on expert consensus annotations. The Jaccard similarity index (intersection over union) between the consensus annotations and the expert annotations was 0.85.*

MT Page 21

Usage of consensus annotations and expert annotations as ground truth to tune the LMP (starfish’s LocalMaxPeakfinder) stringency parameter, which controls the intensity cutoff to detect a peak, resulted in similar precision vs. stringency trends, as well as recall vs. stringency trends (Figure 5). The optimal stringency parameter found using consensus annotations from crowdsourced workers alone as ground truth resulted in a lower precision and slightly higher recall (89.4% and 95.4% respectively, compared with 94.3% and 94.8% from using expert annotations alone as ground truth – 5.3% and 0.63% difference respectively) for RCA image ISS_rnd0_ch1_z0, which contains 1236 spots. These results reflect the fact that some of the spots annotated by the worker consensus were not annotated by the more conservative expert.

MT Page 22, Figure 5 caption

***Figure 5.*** *The optimal “stringency” parameter for starfish’s LocalMaxPeakfinder (LMP) with Rolling Circle Amplification (RCA) images from the starfish database resulted in lower precision and slightly higher recall (89.4% and 95.4% respectively) for RCA image ISS_rnd0_ch1_z0, which contains 1236 spots, when consensus annotations from crowdsourced workers alone were used as ground truth for parameter tuning, compared with 94.3% and 94.8% precision and recall which were achieved when expert annotations alone were used as ground truth for parameter tuning. The optimal stringency parameter found using consensus annotations from crowdsourced worker annotations pooled together with the expert’s annotations resulted in precision and recall almost equivalent to the results using consensus annotations from crowdsourced workers alone (89.5% and 95.3% – 0.1% percent difference for both).*

MT Page 22

S9 Text shows that the training behavior of the expert and crowdsourced annotations is very similar. That is, performance converges to roughly the same level and at roughly the same rate. When blob detection was executed on RCA image ISS_rnd0_ch1_z0, which contains 1236 spots, fifteen ground truth annotations were enough to get 99.1% and 98.1% of the maximum precision performance when the annotations were produced by experts and worker consensus, respectively.

SI Page 16, S17 Figure caption

***S17 Figure. Training behavior was very similar between the expert and consensus annotations.*** *Moreover, when blob detection was executed on ISS_rnd0_ch1_z0, an RCA image from the Starfish dataset that contains 1236 spots, only about 15 or 20 ground truth annotations were needed in order to assure sufficient coverage across the range of spot sizes and intensities and thus get reliable spot size parameters, regardless of whether parameters had been extracted from either expert or consensus ground truth annotations. Above 15 or 20 ground truth annotations, using more sample spots did not significantly improve precision and recall percentage, which leveled off in the high eighties and mid nineties respectively.*

SI Page 23, S9 Text.

Moreover, when blob detection was executed on RCA image ISS_rnd0_ch1_z0, which contains 1236 spots, 15 ground truth annotations were enough to get 99.1% and 98.1% of the maximum precision performance when the annotations were produced by experts and worker consensus, respectively.

**R#2 C2:** *There is a statement in the manuscript ‘The figures and data for this project are available from* [*https://github.com/czbiohub/instapaper.*](https://github.com/czbiohub/instapaper.)*’ As written, I expected to find the input images used at this location, but that does not seem to be the case. I was able to find the input images in other of the other repos. It would be helpful if the authors could clarify this.*

We thank the reviewer for pointing out this need for clarification. This is our revised Data Availability Statement:

The iPython notebooks, input images, and *In Situ* Transcriptomics Annotation (INSTA) pipeline software are available from <https://github.com/czbiohub/instapipeline>. The SpotImage software is available from <https://github.com/czbiohub/spotimage>. The files and iPython notebooks used to generate the figures for this paper are available from <https://github.com/czbiohub/instapaper>.
